# The way is the goal: how SecA transports proteins across the cytoplasmic membrane in bacteria

**DOI:** 10.1093/femsle/fny093

**Published:** 2018-04-12

**Authors:** Tamar Cranford-Smith, Damon Huber

**Affiliations:** Institute for Microbiology and Infection School of Biosciences University of Birmingham Edgbaston Birmingham B15 2TT, UK

**Keywords:** SecA, SecYEG, protein translocation, posttranslational translocation, protein targeting, bacterial secretion

## Abstract

In bacteria, translocation of most soluble secreted proteins (and outer membrane proteins in Gram-negative bacteria) across the cytoplasmic membrane by the Sec machinery is mediated by the essential ATPase SecA. At its core, this machinery consists of SecA and the integral membrane proteins SecYEG, which form a protein conducting channel in the membrane. Proteins are recognised by the Sec machinery by virtue of an internally encoded targeting signal, which usually takes the form of an N-terminal signal sequence. In addition, substrate proteins must be maintained in an unfolded conformation in the cytoplasm, prior to translocation, in order to be competent for translocation through SecYEG. Recognition of substrate proteins occurs via SecA—either through direct recognition by SecA or through secondary recognition by a molecular chaperone that delivers proteins to SecA. Substrate proteins are then screened for the presence of a functional signal sequence by SecYEG. Proteins with functional signal sequences are translocated across the membrane in an ATP-dependent fashion. The current research investigating each of these steps is reviewed here.

## Teaser

The authors review current research into how proteins are recognised and delivered for SecA-mediated translocation in bacteria.

## INTRODUCTION

**Figure 1.**
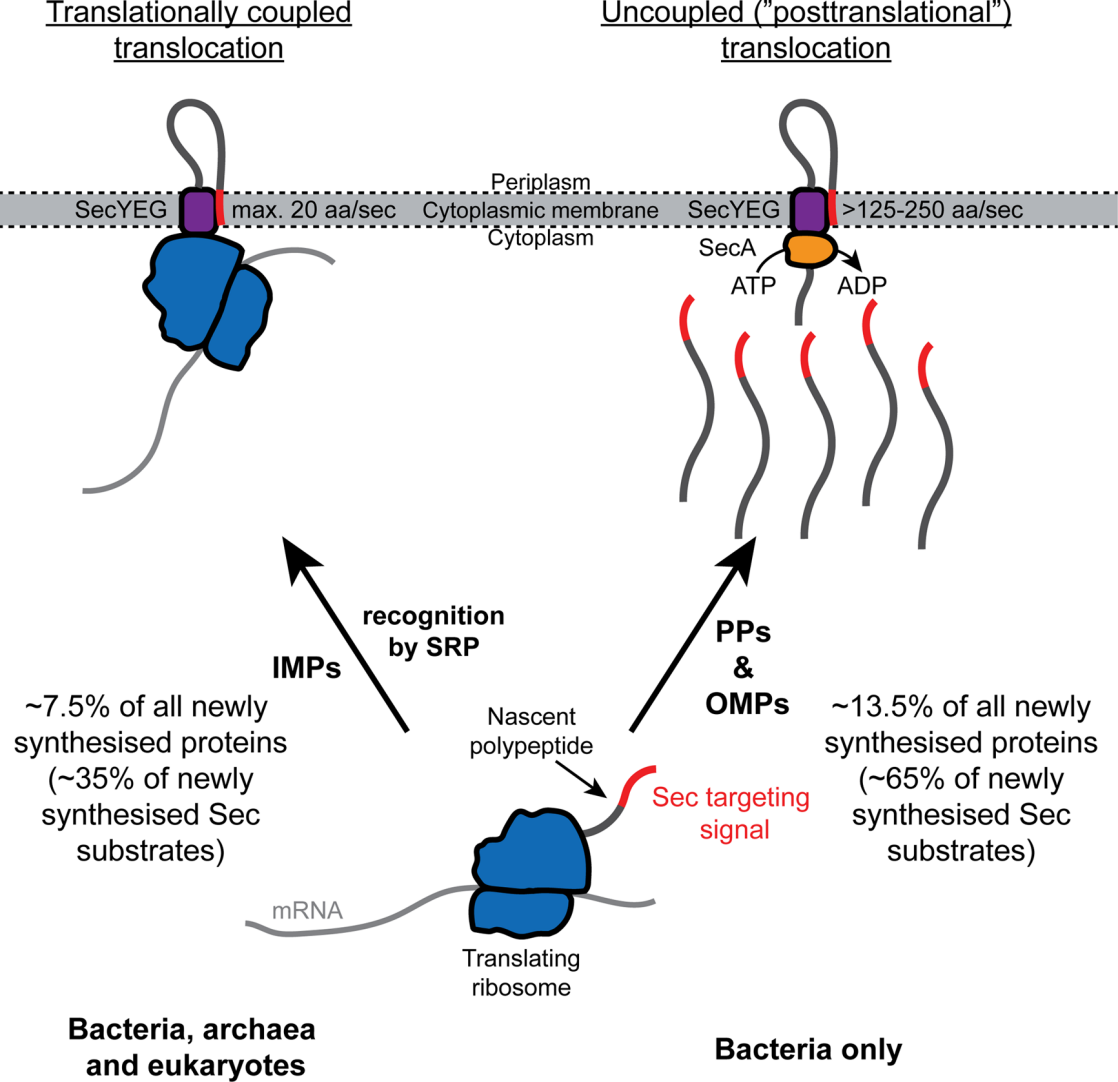
Diagram illustrating the two different pathways for Sec-dependent translocation in *E. coli*. Approximately 20% of all proteins synthesised in *E. coli* are ultimately translocated across the membrane by the Sec machinery. A minority of these newly synthesised proteins (∼7.5%) are integral cytoplasmic membrane proteins (IMPs), most of which are thought to be inserted into the membrane in a translationally coupled fashion (left). The rate of insertion of these proteins is ultimately limited by the rate of translation elongation (∼20 amino acids/s). A much larger fraction of newly synthesised Sec substrates (∼13.5% of all proteins synthesised) are translocated across the membrane by a bacteria-specific mechanism, which is dependent on the ATPase SecA (right). The rate of SecA-mediated translocation is uncoupled from protein synthesis and is much faster (>125 amino acids/second) than the rate of translation elongation (∼20 amino acids/second), which could allow the simultaneous synthesis of multiple substrate proteins destined for the same SecYEG channel.

In bacteria, Sec-dependent translocation of proteins across the cytoplasmic membrane can occur by two different mechanisms: (i) a translationally coupled mechanism, which is conserved in all organisms and which is mediated by the signal recognition particle (SRP), and (ii) a bacteria-specific mechanism that is uncoupled from protein synthesis, which is mediated by the ATPase SecA (Fig. 1). In the translationally coupled pathway, recognition of nascent Sec substrates by the SRP ultimately results in binding of the ribosome to the protein-conducting channel in the cytoplasmic membrane (SecYEG), such that the nascent substrate protein is effectively synthesised directly across (or inserted directly into) the membrane (Saraogi and Shan 2014). If a Sec substrate protein is not recognised by the SRP, it is targeted for translocation by the SecA-mediated pathway (Lee and Bernstein 2001; Schierle *et al.*^2003^). In this pathway, translocation of substrate proteins is independent of (i.e. ‘uncoupled from’) protein synthesis (Josefsson and Randall 1981a,b; Randall 1983). In *Escherichia coli*, a substantial proportion of the proteome is dependent on the SecA-mediated pathway for localisation, including most outer membrane proteins (OMPs) and soluble periplasmic proteins (PPs) (Oliver and Beckwith 1981, 1982a,b; Huber *et al.*2005a). The near universal conservation of SecA in other bacteria suggests that this pathway is similarly important in all bacteria (van der Sluis and Driessen 2006).

SecA-mediated translocation can be divided into two steps: (i) targeting of substrate proteins to the membrane-bound translocation machinery and (ii) translocation through SecYEG across the membrane. Research over the past several years has significantly advanced our understanding of mechanism of both of these steps. Because several recent reviews have focussed on the mechanism of translocation (Park and Rapoport 2012; Chatzi *et al.*^2013^; Collinson, Corey and Allen 2015), this review focuses more closely on the steps preceding translocation across the membrane.

### Why do bacteria have a SecA-mediated translocation pathway?

One perennial questions is: Why do bacteria have two translocation pathways? Although there is not a definitive answer to this question, one potential reason is that bacteria contain a limited number of SecYEG channels. Most bacteria do not extensively invaginate their cytoplasmic membranes or form subcellular compartments dedicated to protein section (e.g. the endoplasmic reticulum of eukaryotes). In addition, the bacterial Sec machinery shares the cytoplasmic membrane with a host of other machineries (e.g. the respiratory chain, cytochromes, F-ATPases, small-molecule transporters, flagella, other secretory systems, etc). Estimates of relative protein abundance from protein synthesis rates suggest that there are not enough channels to support translocation exclusively by the translationally coupled mechanism (Li *et al.*^2014^). Approximately 21% of all newly synthesised proteins are PPs, OMPs or integral cytoplasmic membrane proteins (IMPs) (Li *et al.*^2014^). Assuming that an exponentially growing *E. coli* cell contains around 50 000 ribosomes (Bremer and Dennis 2008), stoichiometric ratios of proteins derived from ribosome profiling experiments suggest that there are ∼5000 SecY molecules per cell (Li *et al.*^2014^). Some studies have estimated that there are as few as 500 copies of SecY per cell, but the number of ribosomes per cell in these instances is proportionately smaller (Matsuyama, Akimaru and Mizushima 1990; Wang *et al.*^2015^). Numbers derived from Li *et al.* (2014) also suggests that there are ∼1.8 million Sec substrate proteins (IMPs, PPs and OMPs) with a combined length of around 420 million amino acids. If translocation were purely cotranslational, all copies of SecY in the cell would be occupied and would need to translocate around 50 amino acids per second at standard rates of growth (generation time of 25–30 min), i.e. more than double the maximum rate of translation elongation (Bremer and Dennis 2008). Thus, cotranslational translocation cannot likely keep pace with the rate of production of Sec substrate proteins. Other ‘back-of-the-envelope’ calculations have yielded similar conclusions (Pugsley 1993; Collinson, Corey and Allen 2015).

However, if translocation is divorced from protein synthesis, multiple ribosomes could simultaneously synthesise proteins that are destined for translocation through the same SecYEG channel (Fig. 1). If translocation is much faster than translation elongation, translocation of all of these proteins could be accomplished in the same amount of time it would take to translocate a single protein cotranslationally. For example, assuming only integral membrane proteins (IMPs) are inserted cotranslationally (Ulbrandt, Newitt and Bernstein 1997; Schibich *et al.*^2016^), ∼3700 SecYEG channels (i.e. ∼75% of the total) would be needed to insert all IMPs, and the rate of cotranslational insertion would be about 30 amino acids per second—much closer to the rate of translation elongation *in vivo* (Li *et al.*^2014^). If the remaining ∼1300 SecYEG channels are left to translocate ∼1.2 million PPs and OMPs per generation (with a total of around 230 million amino acids), the minimum rate of SecA-mediated translocation would be ∼125 amino acids per second. If the channel is dimeric during SecA-mediated translocation (see discussion under ‘the SecYEG complex’ below), this number would be closer to 250 amino acids per second.

### The Sec machinery

#### The SecYEG complex

**Figure 2.**
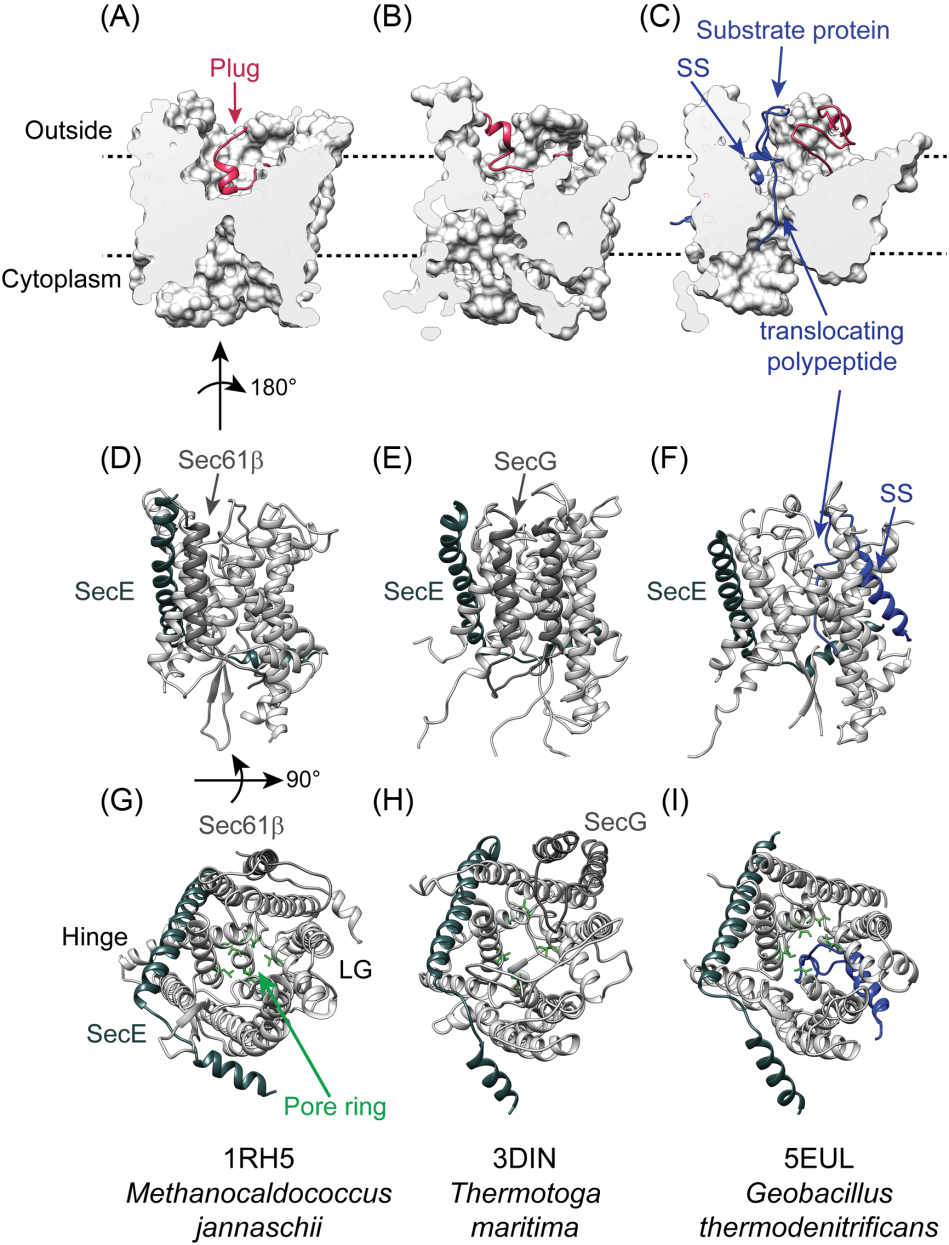
Structure of the Sec translocation channel. The structures of SecYEβ from *Methanocaldococcus jannaschii* in the closed conformation (1RH5; **A**, **D**, **G**) (Van den Berg *et al.*^2004^), SecYEG from *Thermotoga maratima* in the SecA-bound, open conformation (3DIN; **B**, **E**, **H**) (Zimmer, Nam and Rapoport 2008) and SecYE from *Geobacillus thermodenitrificans* in the open, translocating conformation (3EUL; **C**, **F**, **I**) (Li *et al.*^2016^). (**A**–**C**) Cross section of the main body of the channel viewed from the membrane. This representation depicts opening of the constriction in the open conformation and displacement of the plug (crimson, ribbon depiction). In 3EUL, translocating polypeptide (blue, ribbon) can be seen threaded through the constriction, and the attached signal sequence bound in the signal sequence binding site on the exterior of the channel. (**D**–**I**) Ribbon representation of the structural models viewed from the side with the hinge on the left and the lateral gate on the right or (**D**–**F**) from the cytoplasmic face of the membrane. The locations of SecE and SecG (or Sec61β) are indicated. (**G**–**I**) The location of the lateral gate (LG) and hinge are indicated, and the aliphatic residues lining the pore ring constriction are depicted as sticks (green). The translocating peptide is coloured blue (**F** and **I**). The structure of SecA in the 3DIN and 5EUL structures has been cut away to more clearly illustrate the conformational changes in the channel. Structural models were rendered using UCSF-Chimera v 1.12 (Pettersen *et al.*^2004^).

The central component of the Sec machinery is the evolutionarily conserved protein conducting channel in the cytoplasmic membrane (Park and Rapoport 2012). In bacteria, this channel is formed by an integral membrane protein complex composed of SecY, SecE and SecG, which are present in a 1:1:1 stoichiometry. The main component, SecY, is homologous to the eukaryotic Sec61α and archaeal SecY proteins (Park and Rapoport 2012; Collinson, Corey and Allen 2015). SecY contains 10 transmembrane domains arranged in pseudo-2-fold symmetry, which forms an aqueous channel in the cytoplasmic membrane that is shaped like an hourglass (Fig. 2A–F). At the centre of the hourglass, there is a narrow constriction that is lined by long-chain aliphatic residues (Van den Berg *et al.*^2004^) (Fig. 2G–I). Substrate proteins pass through this constriction during translocation across the membrane (Cannon *et al.*^2005^; Li *et al.*^2016^) (Fig. 2C, F and I). In the resting state, the channel is blocked from the exterior by a small α-helical ‘plug’ domain (Van den Berg *et al.*^2004^; Li *et al.*^2007^) (Fig. 2A, D and G). Finally, SecY contains a lateral gate between the halves of the protein, which opens to allow partitioning of transmembrane helices and signal sequences into the membrane (Li *et al.*^2016^) (Fig. 2G). Opposite the lateral gate is a ‘hinge’ that links the two halves of SecY (Van den Berg *et al.*^2004^). SecE and SecG appear to stabilise SecY. SecE binds to the exterior of SecY spanning both sides of the hinge (Van den Berg *et al.*^2004^) (Fig. 2D and G), and SecY is rapidly degraded in its absence (Taura *et al.*^1993^). SecG is not essential for translocation (Brundage *et al.*^1990^; Nishiyama, Hanada and Tokuda 1994), but mutations disrupting SecG decrease the rate of translocation (Nishiyama, Hanada and Tokuda 1994). In addition, genetic evidence suggests that it stabilises the non-translocating form of the channel (Belin *et al.*^2015^).

Structural and biophysical studies suggest that SecYEG occupies at least three conformations: (i) closed, (ii) partially open and (iii) open (Van den Berg *et al.*^2004^; Zimmer, Nam and Rapoport 2008; Allen *et al.*^2016^; Li *et al.*^2016^). In the closed, non-translocating state, the plug domain blocks the exterior opening to the constriction, and the lateral gate is tightly closed (Van den Berg *et al.*^2004^) (Fig. 2A, D and G). Single-molecule fluorescence measurements suggest that binding of ADP-bound SecA to SecYEG results in a small increase in the diameter of the channel, resulting in formation of the ‘part-open’ conformation (Allen *et al.*^2016^). However, binding to ATP causes a large dilation of the constriction and a partial destabilisation of the plug (Zimmer, Nam and Rapoport 2008; Allen *et al.*^2016^) (Fig. 2B, E and H). Opening of the channel could be further stabilised during translocation by the intercalation of a signal sequence or transmembrane helix into a binding site on the exterior of the lateral gate (Li *et al.*^2016^) (Fig. 2F and I). Mutations known as *prl* mutations (Bieker, Phillips and Silhavy 1990) have been isolated in the genes encoding all three components of the channel. These mutations allow the translocation of proteins with defective (or absent) signal sequences *in vivo* (Bieker, Phillips and Silhavy 1990) and appear to destabilise the closed form of the channel (Van den Berg *et al.*^2004^; Li *et al.*^2007^; Belin *et al.*^2015^).

Early studies suggested that the channel is very narrow. Folding, even of relatively small substrate proteins, prevents translocation across the membrane *in vivo* (Randall and Hardy 1986), and the introduction of stably folded elements into a Sec substrate protein results in trapping of partially translocated intermediates *in vitro* (Uchida, Mori and Mizushima 1995). However, the exact size of the channel formed by SecYEG is a matter of some debate. Molecular dynamics simulations of the SecYEG monomer suggest that it could accommodate structures up to ∼16 Å (Gumbart and Schulten 2006, 2007; Tian and Andricioaei 2006), and experimental evidence suggests that the channel can expand to ∼22 Å (Bonardi *et al.*^2011^).

Several lines of evidence suggest that SecYEG normally dimerises, but the physiological role of the dimers, if any, is unknown. The most widely accepted dimer interface is located at the back of the hinge domain, resulting a ‘back-to-back’ arrangement (Veenendaal, van der Does and Driessen 2001; Mori *et al.*^2003^; Deville *et al.*^2011^). High-resolution structures suggest that one copy of SecYEG interacts with SecA during translocation (Zimmer, Nam and Rapoport 2008; Li *et al.*^2016^). However, conclusions from mechanistic studies investigating the requirement for dimerisation of SecYEG in SecA-mediated translocation are mixed (Osborne and Rapoport 2007; Deville *et al.*^2011^). Furthermore, it has been proposed that SecYEG could (also) form ‘front-to-front’ dimers, in which SecYEG protomers interact with each other *via* the lateral gate, in order to accommodate substrate proteins with more extensive tertiary structure (Bonardi *et al.*^2011^; Das and Oliver 2011). However, a front-to-front dimer would preclude the interaction of SecY with many auxiliary Sec components (see below), which appear to interact with the lateral gate (Sachelaru *et al.*^2013^, ^2014^; Botte *et al.*^2016^).

#### SecA

**Figure 3.**
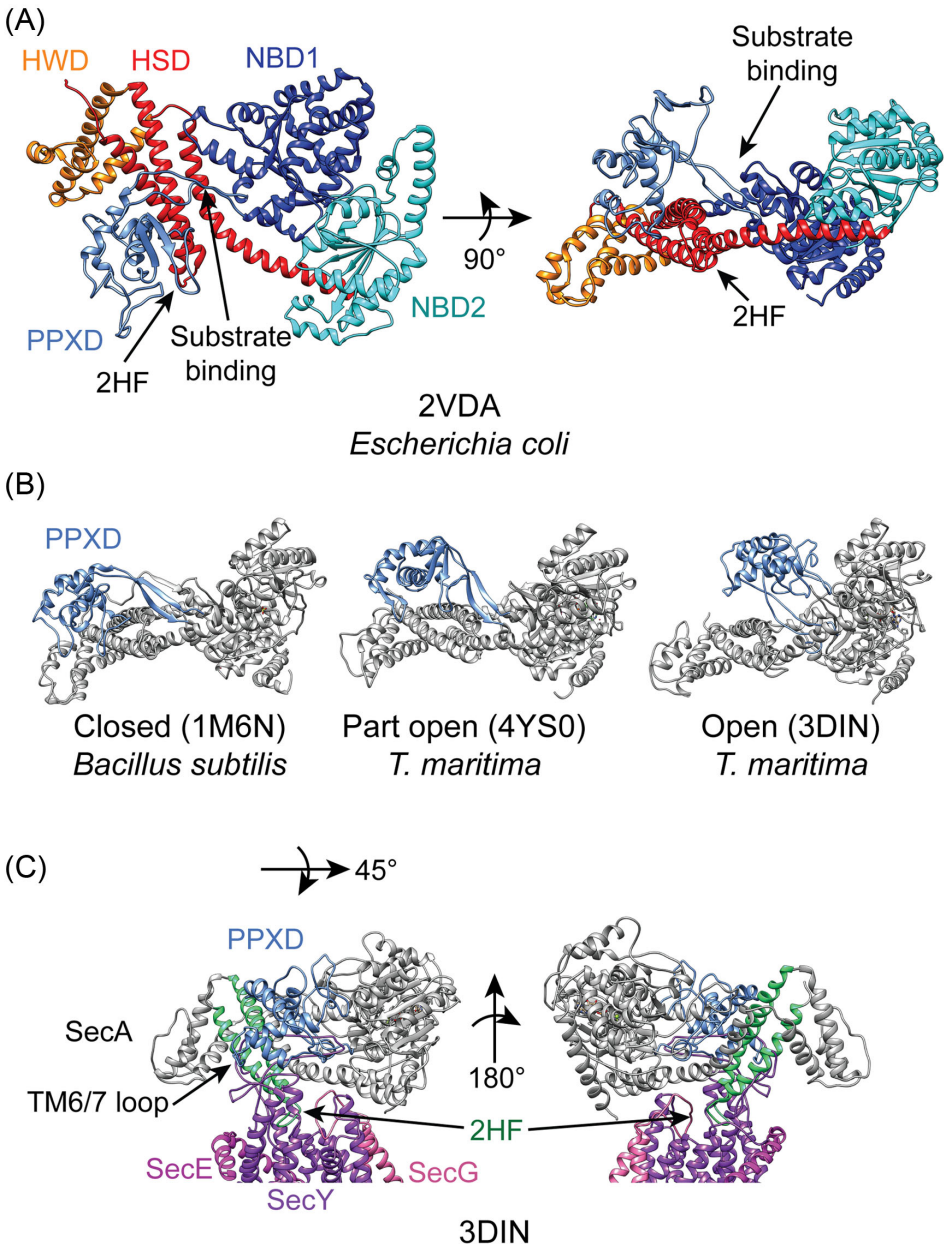
Structure of SecA and conformational changes in the PPXD. (**A**) NMR structure of SecA from *E. coli* (2VDA) viewed from two different angles (Gelis *et al.*^2007^). The locations of NBD-1 (dark blue), NBD-2 (cyan), PPXD (light blue), HSD (red) and HWD (orange) are indicated. The CTT is absent in high-resolution structures and is not depicted. The approximate locations of the substrate binding site between the PPXD and NBD-2 and the signal sequence binding site between the PPXD and the HWD are likewise indicated. (**B**) Structures of SecA illustrating the large translational and rotational movement of the PPXD (light blue) during conversion between the closed (1M6N) (Hunt *et al.*^2002^), part open (4YS0) (Chen *et al.*^2015^) and open (3DIN) (Zimmer, Nam and Rapoport 2008) conformations. (**C**) Structure of SecA (grey, PPXD in light blue) in complex with SecYEG (purple) (3DIN) from two angles (Zimmer, Nam and Rapoport 2008). This structure illustrates the deep penetration of the two-helix finger (2HF; green) into the SecYEG channel (purple) and binding of the TM6/7 loop by the PPXD of SecA. Structural models were rendered using UCSF-Chimera v 1.12 (Pettersen *et al.*^2004^).

SecA is required for the translocation of most proteins in *E. coli* (Oliver and Beckwith 1981, 1982a,b). The ATPase activity of SecA occurs at the interface of two nucleotide-binding domains (NBD-1 and NBD-2) (Schmidt *et al.*^1988^; Lill *et al.*^1989^; Hunt *et al.*^2002^) (Fig. 3A, dark blue and cyan, respectively), which are related to those of RecA-like helicases (Hunt *et al.*^2002^; Sharma *et al.*^2003^; Ye *et al.*^2004^). The primary structure of NBD-1 is interrupted by a domain known as the polypeptide crosslinking domain (PPXD) (Hunt *et al.*^2002^) (Fig. 3A, light blue), which contacts the substrate polypeptide during translocation (Bauer and Rapoport 2009). C-terminal to NBD-2 is an α-helical domain that is composed of two subdomains (Hunt *et al.*^2002^): (i) the α-helical scaffold domain (HSD) (Fig. 3A, red) and (ii) the α-helical wing domain (HWD) (Fig. 3A, orange). The HSD contains a two-helix finger (2HF) near the C-terminus, which contacts the substrate protein and plays a critical role in protein translocation (Erlandson *et al.*^2008^). In addition, most SecA proteins contain a C-terminal tail (CTT) that is not resolved in high-resolution structures. In *E. coli*, the CTT contains a small zinc-binding domain (ZnBD) that is required for the efficient interaction of SecA with its binding partner SecB (Fekkes *et al.*^1997^, ^1999^). However, the ZnBD is present in SecA in many species that lack SecB (van der Sluis and Driessen 2006), suggesting that the ZnBD (and the CTT) has another function. For example, it has been suggested that the CTT could autoinhibit SecA by competing for interaction with substrate proteins although the significance of this activity is unknown (Gelis *et al.*^2007^).

SecA undergoes a large conformational change upon interaction with substrate protein or SecYEG (Zimmer, Nam and Rapoport 2008; Chen *et al.*^2015^) (Fig. 3B). In the x-ray crystal structure of SecA from *Bacillus subtilis* (1M6N), the PPXD is positioned near the HWD (Hunt *et al.*^2002^), a state known as the ‘closed’ conformation. However, upon interaction with substrate protein or SecYEG, the PPXD undergoes a large rotation and translation to bring it into proximity of NBD2 (and away from the HWD) (Zimmer, Nam and Rapoport 2008; Chen *et al.*^2015^)—i.e. the ‘open’ conformation. SecA binds to substrate protein in the groove between NBD-1/-2 and the PPXD (Zimmer and Rapoport 2009). ‘Opening’ of the clamp encloses the substrate protein, which is thought to stabilise its interaction with SecA (Zimmer, Nam and Rapoport 2008; Gold *et al.*^2013^). Opening of the clamp also activates the ATPase activity of SecA by increasing the rate of nucleotide exchange (Fak *et al.*^2004^; Gold *et al.*^2013^). The PPXD occupies several part-open conformations in different high resolution structures (Gelis *et al.*^2007^; Chen *et al.*^2015^). For example, NMR studies suggest that ∼10% of the protein is in the closed conformation while ∼90% occupies a ‘partially open’ conformation (Gelis *et al.*^2007^). These intermediate conformations probably represent transition states between the closed and open conformations but could serve another as-yet undetermined function.

Binding of SecA to SecYEG involves extensive contact between the two proteins, and results in conformational changes in both proteins (Mori and Ito 2006; Zimmer, Nam and Rapoport 2008; Das and Oliver 2011; Li *et al.*^2016^) (Fig. 3C). For example, the 2HF of SecA inserts deep into the channel, and SecA binds the large cytoplasmic TM6/7 loop of SecY between the PPXD and the HSD (Zimmer, Nam and Rapoport 2008). Binding of SecA to ATP appears to destabilise the closed form of the channel (Zimmer, Nam and Rapoport 2008; Allen *et al.*^2016^; Li *et al.*^2016^).

The oligomeric state of SecA during translocation has been a matter of some dispute. It has been well noted that SecA forms homodimers in solution (Akita *et al.*^1991^; Driessen 1993; Hirano, Matsuyama and Tokuda 1996; Doyle, Braswell and Teschke 2000; Woodbury, Hardy and Randall 2002). X-ray crystal structures of the SecA dimer suggest several different dimer interfaces (for example, see Hunt *et al.*^2002^; Vassylyev *et al.*^2006^; Zimmer, Li and Rapoport 2006; Papanikolau *et al.*^2007^). Site-specific crosslinking studies indicate that SecA prefers one of these conformations when overproduced *in vivo* (Banerjee, Lindenthal and Oliver 2017). However, purified SecA probably populates several different dimers in solution (Woodbury, Hardy and Randall 2002; Kusters *et al.*^2011^; Auclair, Oliver and Mukerji 2013). The role of this dimer (if any) is unclear. Dimerisation appears to enhance protein translocation (Driessen 1993; Jilaveanu, Zito and Oliver 2005; Jilaveanu and Oliver 2006; Kusters *et al.*^2011^; Gouridis *et al.*^2013^). However, monomeric versions of SecA can promote protein translocation (Or, Navon and Rapoport 2002; Or *et al.*^2005^), and high-resolution structures of the SecA-SecYEG complex indicate that SecA docks with SecYEG in a 1:1 stoichiometry (Zimmer, Nam and Rapoport 2008; Li *et al.*^2016^).

### Sec targeting signals

Proteins destined for SecA-mediated translocation across the membrane share two common features. First, they contain an internally encoded targeting signal that allows them to be recognised by the Sec machinery, which usually takes the form of an N-terminal signal sequence (Hegde and Bernstein 2006). Second, all substrate proteins contain features which allow them to be maintained in an unfolded conformation prior to translocation (Randall and Hardy 1986; Schatz and Dobberstein 1996).

#### N-terminal signals sequences

**Figure 4.**
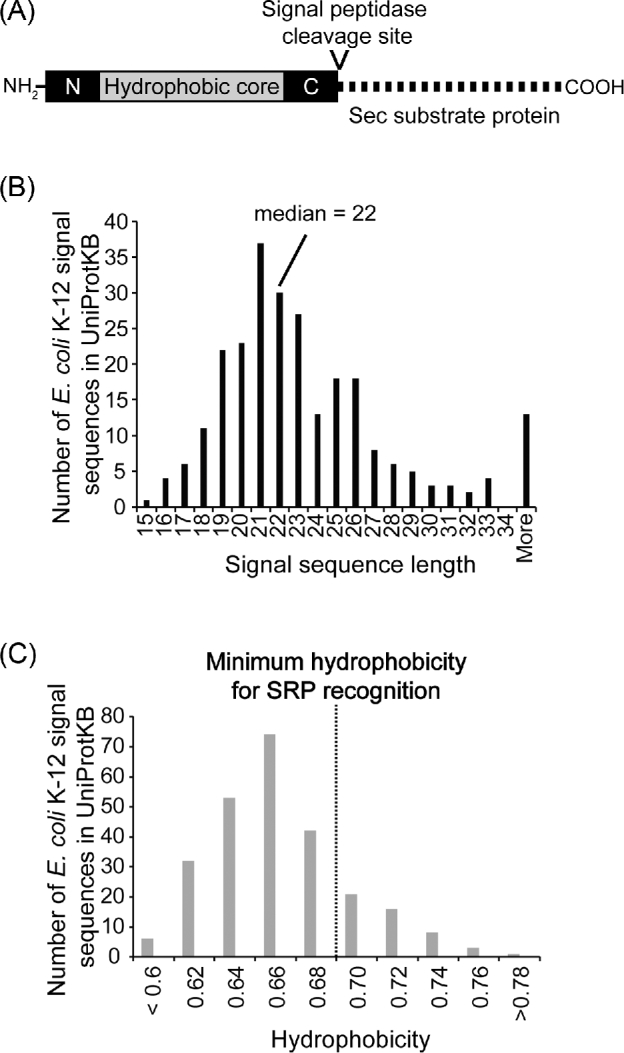
Properties of *E. coli* K-12 signal sequences. (**A**) Diagram of the primary structure of an N-terminal signal sequence, including the N-domain (N; black), the hydrophobic core (grey) and the C-domain (C; black). If present, the signal peptidase recognition site is contained at the C-terminal portion of the C-domain and results in cleavage of the signal sequence from the mature Sec substrate protein during translocation. (**B**) Analysis of the length of *E. coli* signal sequences in the UniProtKB database. Protein entries in the UniProtKB database for *E. coli* K-12 were screened for those containing the key feature ‘signal peptide’. The lengths of these signal peptides were then determined and plotted as a histogram. The median signal sequence length of this set (22) is indicated. (**C**) The hydrophobicity of the signal sequences in (B) was determined according to Huber *et al.* (2005a) and plotted as a histogram according to their hydrophobicity. The minimum hydrophobicity required for SRP recognition indicates that most N-terminal cleavable signal sequences are targeted for SecA-mediated translocation.

In the 1970s, Blobel and colleagues proposed that secreted proteins contained peptide sequences at their N-termini that allowed them to be recognised by the translocation machinery (Blobel and Dobberstein 1975a,b). These N-terminal signal sequences were first identified genetically in bacteria by mutations that prevented translocation of reporter fusion proteins across the cytoplasmic membrane (Emr, Schwartz and Silhavy 1978; Bassford and Beckwith 1979). Subsequent work indicated that signal sequences have a conserved primary structure, which consists of a hydrophobic core flanked by shorter N- and C-domains (Fig. 4A) (von Heijne 1990; Hegde and Bernstein 2006). The N-domain, located N-terminal to the hydrophobic core, is positively charged and may play a role in orienting the signal sequencing in the membrane (von Heijne 1990; Andersson, Bakker and von Heijne 1992). The hydrophilic C-domain is less positively charged than the N-domain and contains a recognition site for signal peptidase, which allows cleavage of the signal sequence from the precursor to form the mature protein (Perlman and Halvorson 1983; Wolfe and Wickner 1984; von Heijne 1990; Hegde and Bernstein 2006). The C-domain also contains a recognition site for signal peptidase-1 or -2, which remove the signal sequence from the mature protein during translocation (Josefsson and Randall 1981a,b; Hegde and Bernstein 2006). Processing by signal peptidase-2 also results in lipidation of the N-terminal cysteine (Hegde and Bernstein 2006). All three domains can vary in length. However, an analysis of signal sequence-containing proteins from *E. coli* K-12 in the UniprotKB database indicates that the median signal sequence length in *E. coli* is 22 amino acids, with a minimum of 15–16 amino acids (Fig. 4B).

The decision whether to export substrate proteins by the translationally coupled pathway or by the translationally uncoupled pathway depends on the hydrophobicity of the signal sequence (Lee and Bernstein 2001; Bowers, Lau and Silhavy 2003; Schierle *et al.*^2003^; Huber *et al.*2005a). Very hydrophobic signal sequences are recognised by the SRP and are targeted for translationally coupled translocation (Huber *et al.*2005a; Schibich *et al.*^2016^). Those that fail to be recognised by the SRP appear to be targeted to the SecA-dependent pathway by default (Lee and Bernstein 2001; Schierle *et al.*^2003^).

#### Other targeting signals

Some Sec substrate proteins appear to contain additional targeting signals that allow them to be recognised by the Sec machinery. For example, it has been suggested that the molecular chaperone SecB can recognise a subset of Sec substrate proteins (Derman *et al.*^1993^; Kumamoto and Francetic 1993; Prinz *et al.*^1996^; Randall *et al.*^1997^, ^1998^; Knoblauch *et al.*^1999^). In addition, recent work suggests that SecA can recognise polypeptide sequences in the mature portion of some substrate proteins (Chatzi *et al.*^2017^). Finally, at least one protein (the SodA protein of *Rhizobium leguminosarum*), which lacks a signal sequence, can be recognised by the Sec machinery in both *Rhizobium* and *E. coli* (Krehenbrink, Edwards and Downie 2011).

#### Folding of the substrate protein

Because the channel is only large enough to accommodate unfolded proteins (Van den Berg *et al.*^2004^; Gumbart and Schulten 2006, 2007; Tian and Andricioaei 2006; Bonardi *et al.*^2011^; Li *et al.*^2016^), Sec substrate proteins must be kept unfolded in the cytoplasm (Randall and Hardy 1986; Teschke *et al.*^1991^; Uchida, Mori and Mizushima 1995; Li *et al.*^2016^). Mutations that slow folding can compensate for defects in targeting caused by defective signal sequences (Liu *et al.*^1988^; Teschke *et al.*^1991^; Song and Park 1995). Indeed, many proteins (e.g. some normally cytoplasmic proteins and heterologously expressed proteins) are refractory to translocation because they rapidly fold in the cytoplasm before they can be transported through SecYEG (Huber *et al.*2005b, 2010; Steiner *et al.*^2006^). Because most substrates of the SecA-mediated pathway exist transiently as full-length cytoplasmic intermediates (Josefsson and Randall 1981a,b), bacteria have evolved multiple mechanisms to prevent premature cytoplasmic folding. For example, the signal sequences of some precursor proteins can slow their folding (Liu *et al.*^1988^; Park *et al.*^1988^; Beena, Udgaonkar and Varadarajan 2004). In addition, molecular chaperones (e.g. SecB) can bind to a subset of Sec substrates and prevent cytoplasmic folding (Collier *et al.*^1988^; Kumamoto and Gannon 1988). Finally, some substrate proteins require covalent modifications (e.g. disulfide bonds) in order to fold stably, and these modifications can only be made after the protein has been localised to the correct compartment (Hatahet, Boyd and Beckwith 2014).

### Recognition of substrate proteins by the Sec machinery

Substrate proteins must be recognised by some component of the Sec machinery. For many years, it was generally assumed that SecB recognised substrate proteins and delivered them for SecA-mediated translocation by interacting with SecA (Hartl *et al.*^1990^; Fekkes *et al.*^1998^; Driessen and Nouwen 2008). However, recent research also implicates SecA and SecYEG in substrate protein recognition, and it seems likely that all three components are involved in targeting substrate proteins for SecA-mediated translocation.

#### Recognition by SecB

SecB is a homotetrameric molecular chaperone, which is required for the efficient translocation of a subset of proteins exported by the SecA-mediated pathway (Randall and Hardy 2002). SecB interacts with its nascent substrate proteins cotranslationally (Kumamoto and Francetic 1993), and mutations that disrupt the *secB* gene cause the translocation of maltose-binding protein (MalE) to become fully posttranslational (Kumamoto and Gannon 1988). (A significant portion of newly synthesised MalE is translocated cotranslationally although translocation begins at much longer nascent chain lengths than is typical for SRP-mediated translocation (Josefsson and Randall 1981a,b; Schierle *et al.*^2003^), which is typical of many substrates of the SecA-mediated pathway (Josefsson and Randall 1981a,b)). Biochemical studies supported the idea that substrate proteins were transferred from SecB to SecA and then translocated through SecYEG (Hartl *et al.*^1990^). SecB interacts with SecA (den Blaauwen *et al.*^1997^; Fekkes *et al.*^1997^; Randall and Henzl 2010), and substrate protein strengthens this interaction (Fekkes *et al.*^1998^). Finally, mutant SecB proteins that are defective for interaction with SecA accumulate in a substrate-bound form *in vivo* (Gannon and Kumamoto 1993).

However, SecB cannot be the only, or even the primary, protein that recognises substrates of the SecA-mediated pathway. *Escherichia coli* mutants lacking SecB are viable (Kumamoto and Gannon 1988; Shimizu, Nishiyama and Tokuda 1997) and are defective in the translocation of a relatively small subset of proteins (Kumamoto and Beckwith 1985; Baars *et al.*^2006^). Even for these substrate proteins, translocation is only partially defective in the absence of SecB (Kumamoto and Beckwith 1983). Finally, SecB is not found in all bacteria (van der Sluis and Driessen (2006)).

#### Direct recognition of substrate proteins by SecA

One possibility is that SecA recognises its substrate proteins directly. The only protein components required for translocation *in vitro* are SecA, SecY and SecE (Brundage *et al.*^1990^), suggesting that one of these proteins can recognise substrate proteins. SecA binds directly to signal sequence-like peptides (Gelis *et al.*^2007^; Auclair *et al.*^2010^; Zhang *et al.*^2016^). In addition, the presence of a signal sequence increases the affinity of SecA for unfolded proteins (Kebir and Kendall 2002; Gouridis *et al.*^2009^) and alters the behaviour of SecA towards substrate protein (Eser and Ehrmann 2003). These findings suggest that SecA can directly recognise proteins containing signal sequences. SecA has also been implicated in the recognition of internally encoded targeting signals (Chatzi *et al.*^2017^).

SecA also interacts cotranslationally with nascent Sec substrates *in vivo* (Chun and Randall 1994; Huber *et al.*^2017^). This interaction appears to be mediated by a specific interaction between SecA and the ribosome (Huber *et al.*^2011^, ^2017^). SecA binds to the ribosome near the site where nascent chains emerge into the cytoplasm (Huber *et al.*^2011^; Singh *et al.*^2014^), and disrupting this interaction causes a partial defect in SecA-mediated translocation (Huber *et al.*^2011^). In addition, it strongly disrupts the interaction between SecB and its nascent substrate proteins (Huber *et al.*^2017^), suggesting that the interaction of SecA with nascent substrates precedes the interaction of SecB with these proteins. It is yet not known whether SecA recognises all substrate proteins cotranslationally or only a subset. One recent study found that binding of SecA to the ribosome was required for the insertion of the IMP RodZ (Wang, Yang and Shan 2017). This requirement could explain the dependence of MreB, which binds to RodZ, on SecA for its localisation (Govindarajan and Amster-Choder 2017). However, a different study found that SecA interacted with all (or most) nascent Sec substrates (Huber *et al.*^2017^). One explanation is that SecA normally recognises all substrate proteins cotranslationally but that only a subset require cotranslational recognition for insertion.

#### Recognition of substrate proteins by the lateral gate of the SecYEG channel

Finally, it is possible that the channel itself recognises substrate proteins. It has been suggested that signal sequences are required to ‘unlock’ SecYEG prior to translocation (Hizlan *et al.*^2012^), which could serve as a recognition step. In addition, the presence of a signal-sequence-like peptide can stimulate SecA-mediated translocation of signal sequence-less substrate proteins when added *in trans* (Gouridis *et al.*^2009^), suggesting that SecY itself may recognise substrate proteins. However, direct recognition by SecYEG seems unlikely since SecY would need to screen all newly synthesised substrate proteins, even those synthesised in the cytoplasm.

**Figure 5.**
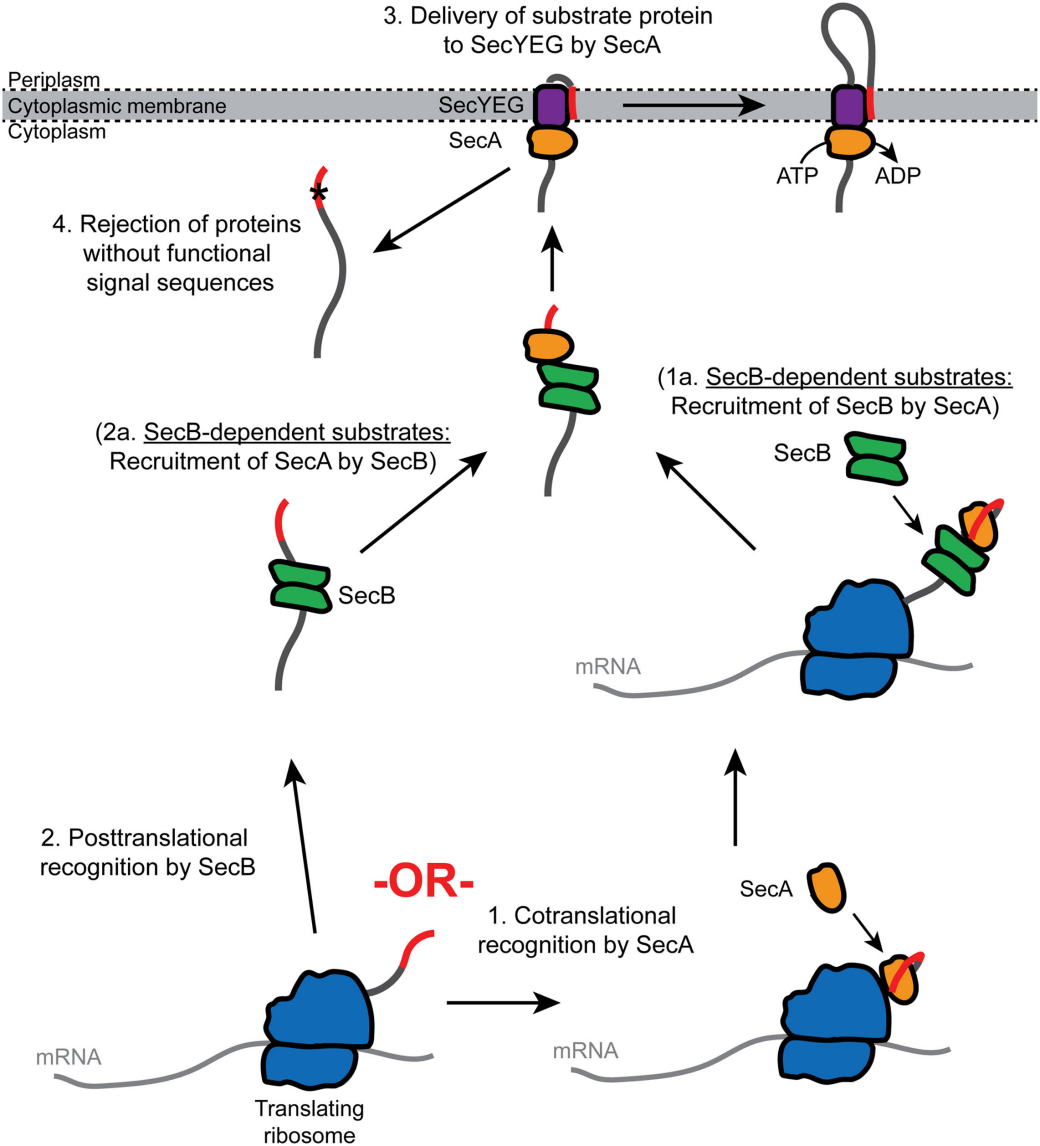
Proposed pathways for targeting substrate proteins for SecA-mediated translocation. It appears that substrate proteins are initially recognised by Sec machinery by two different mechanisms: (1) SecA cotranslationally recognises nascent Sec substrate proteins as they emerge from the ribosome by virtue of an internally encoded targeting signal. SecA may then recruit SecB to the substrate protein (1a) or deliver the protein directly to SecYEG (3). (2) Alternatively, a subset of substrate proteins may be recognised by SecB and delivered to the Sec machinery through the interaction of SecB with SecA (2a), which ultimately delivers the protein to SecYEG (3). Incorporation of the signal sequence into the lateral gate of SecYEG may serve as a final quality control step to prevent the translocation of proteins with signal-sequence-like regions in their primary structure (4).

Together, this research suggests that substrate recognition occurs in two steps: initial recognition and quality control (Fig. 5). First, SecA recognises the substrate protein. Alternatively, molecular chaperones, such as SecB, recognise substrate proteins and deliver them to the translocation machinery by interacting with SecA. Second, interaction of the signal sequence with SecYEG serves as a quality control step ensuring that only proteins with functioning signal sequences are translocated across the membrane.

### The role of ribosome-associated chaperones in determining the timing of translocation

Different substrate proteins are delivered to SecYEG for translocation with differing kinetics *in vivo* (Josefsson and Randall 1981a,b). In *E. coli*, one factor that influences the timing of delivery is the ribosome-associated chaperone Trigger Factor (TF) (Oh *et al.*^2011^). Mutations disrupting the gene encoding TF (*tig*) cause the translocation of multiple SecA substrate proteins to become more cotranslational (Ullers *et al.*^2007^; Oh *et al.*^2011^) and can suppress the translocation defect caused by mutations in *secB* (Lee and Bernstein 2002; Ullers *et al.*^2007^). This phenotype is reminiscent of the ability of chloramphenicol, which causes translocation to become more cotranslational at subinhibitory concentrations (Kadokura and Beckwith 2009), to suppress translocation defects in many *sec* mutants (Lee and Beckwith 1986). The physiological importance of the delay in targeting caused by TF is unknown. One idea is that competition between TF and the SRP influences the choice of translocation pathways, perhaps by making the SRP more selective (Eisner *et al.*^2003^, ^2006^; Bornemann, Holtkamp and Wintermeyer 2014; Ariosa *et al.*^2015^). Alternatively, it is possible that TF prevents excessive cotranslational translocation by the SecA mediated pathway, which is toxic (van Stelten *et al.*^2009^).

### The mechanism of SecA-mediated protein translocation

The mechanism of SecA-mediated translocation has been investigated intensively and is a rich source of mechanistic evidence. SecA can initiate translocation in both the ATP- and ADP-bound forms, and the subsequent translocation of substrate proteins through SecYEG requires rounds of ATP binding and hydrolysis (Schiebel *et al.*^1991^). The rate-limiting step in the ATPase cycle of SecA is nucleotide exchange, and interaction of SecA with substrate protein and with SecYEG increases the rate of nucleotide exchange (Fak *et al.*^2004^). Biochemical studies have suggested that each round of ATP binding and hydrolysis results in the translocation of ∼50 amino acids (Tani *et al.*^1989^; Schiebel *et al.*^1991^; van der Wolk *et al.*^1997^), and the length of time required for translocation increases with increasing length of substrate protein (Tomkiewicz *et al.*^2006^). Binding of SecA to ATP results in high-affinity binding to SecYEG and in the protection of a large portion of SecA from proteolytic digestion, indicating that SecA undergoes a large conformational change upon binding to ATP (Economou and Wickner 1994). At later stages of translocation, the proton-motive force (PMF) also assists in translocation by an unknown mechanism (Schiebel *et al.*^1991^).

Despite this wealth of evidence, the molecular mechanism of SecA-mediated translocation is disputed. Several distinct mechanistic models have been proposed to account for the above observations, and these models can generally be divided into three types: (i) processive, (ii) probabilistic and (iii) mixed processive/probabilistic. Processive models depend entirely on mechanical pushing force provided by SecA (e.g. see Gouridis *et al.*^2013^). In contrast, probabilistic models rely on Brownian movement of the polypeptide chain through a channel (e.g. see Allen *et al.*^2016^). Finally, mixed models contain both processive and probabilistic elements (e.g. see Bauer *et al.*^2014^).

#### Processive translocation by mechanical pushing

**Figure 6.**
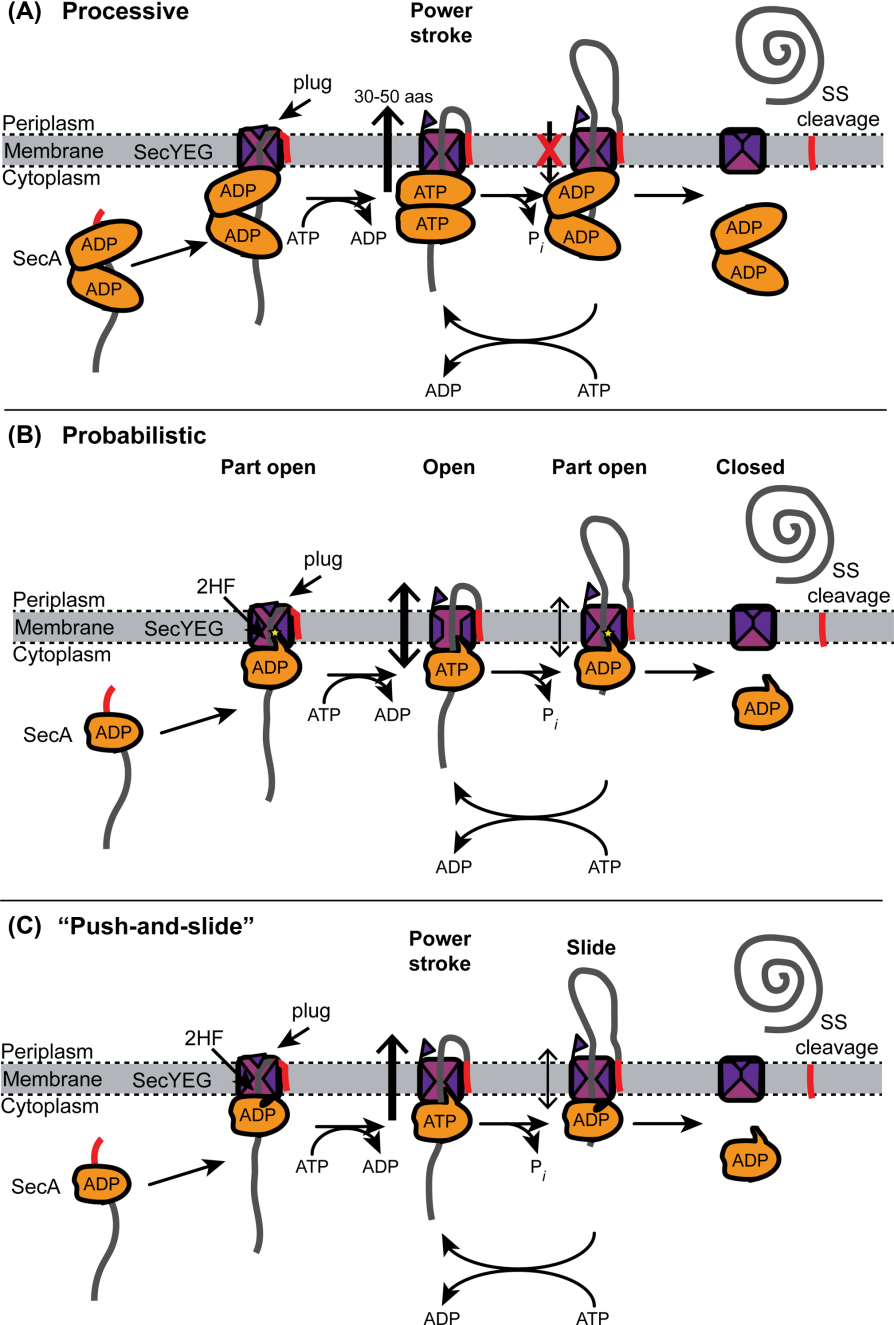
Proposed mechanisms for SecA-mediated translocation. Mechanistic models for translocation can be grouped into three classes: (**A**) processive models, (**B**) probabilistic models (ratched diffusion) and (**C**) mixed processive/probabilistic models (‘push-and-slide’). (A) Processive models require a ‘power stroke’ that results in the translocation of around 5 kDa (∼50 amino acids) per round of ATP binding and hydrolysis. In order to account for the large ‘step size’ for each round of translocation, most processive models require SecA to oligomerise. Binding of SecA to ATP results in a conformational change that mechanically pushes the substrate protein through SecYEG. It has been proposed that hydrolysis of ATP could result in a second pushing step (van der Wolk *et al.*^1997^). (B) In the ratcheted diffusion model (Allen *et al.*^2016^), translocation is probabilistic. SecA gates opening of the channel, allowing the substrate protein to translocation through the channel by diffusion. In the ADP-bound form, SecA causes the channel to occupy a part-open conformation that allows limited diffusion of the polypeptide chain. However, the presence of a polypeptide chain in the channel is sensed by the 2HF of SecA, which promotes nucleotide exchange. Binding to ATP opens the channel and allowing free diffusion of the polypeptide chain. (**C**) In the ‘push-and-slide’ mechanism (Bauer *et al.*^2014^), binding of SecA to the channel results in opening of the channel and allows the polypeptide chain to diffuse freely through it. The direction of diffusion is biased by pushing the 2HF—binding to ATP results in a translocation of the 2HF, which pushes the polypeptide chain through SecYEG. ATP hydrolysis resets the 2HF without pulling on the polypeptide chain.

Traditionally, SecA has been viewed as a mechanical pump that pushes proteins through SecYEG (van der Wolk *et al.*^1997^). In order to translocate substrate proteins in discrete steps of 50 amino acids, processive models would require a movement within SecA of ∼75Å (or two movements of ∼37Å, van der Wolk *et al.*^1997^), assuming that the substrate protein is purely α-helical. Most plausible processive models require SecA to dimerise or to oligomerise in order to account for such large translational motions (Gouridis *et al.*^2013^). Multimerisation would also be consistent with the similarity of SecA to RecA-like helicases, which frequently multimerise in order to unwind RNA molecules (Ye *et al.*^2004^). In these models, binding of SecA to ATP causes a conformational change in the SecA dimer, which pushes the protein through SecYEG (Schiebel *et al.*^1991^) (Fig. 6A). Subsequent hydrolysis of ATP to ADP results in resetting of the motor to the pre-translocation state and could also result in a second round of translocation (van der Wolk *et al.*^1997^). One key feature of processive models is that translocation is unidirectional: some feature prevents the retrograde translocation of the substrate protein when the motor protein resets to its pre-translocation state.

Recent research suggesting that SecA is monomeric during translocation has called into question the validity of most classical processive models (Or, Navon and Rapoport 2002; Or *et al.*^2005^; Zimmer, Nam and Rapoport 2008). However, one recent study suggests that SecA could cycle between dimeric and monomeric forms during translocation (Gouridis *et al.*^2013^). If true, such a mechanism could explain some of the apparently contradictory results surrounding the oligomeric state of the protein.

#### Probabilistic translocation by ratcheted diffusion

An alternative to translocation by mechanical pushing is ratcheted diffusion of the substrate protein. In this model, SecA serves as a regulator protein, which causes opening of the SecYEG channel in the presence of translocating polypeptide (Fig. 6B) (Allen *et al.*^2016^). In summary, binding of SecA to ADP causes SecYEG to occupy a partially open conformation, and binding of SecA to ATP causes SecYEG to occupy the fully open conformation. In the partially open conformation, only amino acids with small side chains can pass through the constriction in SecYEG. However, the presence of a translocating polypeptide in the channel is sensed by SecYEG and the 2HF of SecA and results in exchange of ADP for ATP. Binding to ATP causes SecA to open the channel, allowing the translocation of polypeptides containing bulky amino acids. This model requires a single copy of SecA but is still consistent with the basic parameters of SecA-mediated translocation determined by biochemical experiments. In addition, it provides an explanation for (i) the importance of the 2HF in SecA-mediated translocation (Erlandson *et al.*^2008^; Zimmer, Nam and Rapoport 2008); (ii) how limiting the rate of nucleotide exchange promotes protein translocation (Fak *et al.*^2004^); and (iii) why *prl* mutations, which appear to destabilise the channel, promote more promiscuous translocation (Van den Berg *et al.*^2004^). Finally, a diffusional model could explain the rates of translocation observed *in vivo* (Simon, Peskin and Oster 1992). One issue not addressed by this model is how translocation is driven in the forward direction. It is possible that interaction with periplasmic chaperones, such as PpiD (Antonoaea *et al.*^2008^), prevents backsliding *in vivo*, and several additional mechanisms have been suggested (Allen *et al.*^2016^).

#### The push-and-slide model (mixed processive and probabilistic)

Finally, it has been suggested that translocation could proceed by a ‘push-and-slide’ mechanism, which includes elements of both processive and probabilistic models (Bauer *et al.*^2014^) (Fig. 6C). This mechanism is also diffusional in nature, but the direction of translocation is biased due to pushing by the 2HF. Binding of SecA to SecYEG results in opening of the channel, allowing the movement of polypeptides through the channel. Binding to ATP results in translation of the 2HF. The tip of the 2HF contains a conserved aromatic residue, which is thought to bind to substrate protein and ‘push’ the substrate polypeptide through SecYEG (Erlandson *et al.*^2008^; Bauer *et al.*^2014^). Although this movement is not large, diffusion of the polypeptide through SecYEG allows for the large step (Schiebel *et al.*^1991^; Bauer *et al.*^2014^). Afterwards, hydrolysis of ATP resets the 2HF to its pre-translocation position. This model has many of the same strengths as the ratcheted diffusion model (Allen *et al.*^2016^) and provides an explanation for the overall directionality of translocation. However, it is not clear how the aromatic residue in the 2HF ‘lets go’ of the substrate polypeptide after ATP hydrolysis in order to prevent retrograde translocation. In addition, this model relies on a large translational movement by the 2HF, but biochemical studies suggest that such a movement is not required for translocation (Whitehouse *et al.*^2012^).

### The involvement of the auxiliary Sec components in SecA-mediated translocation

SecYEG associates with several auxiliary Sec components, including SecD, SecF, YajC and YidC, to form a supercomplex known as the holotranslocon (Duong and Wickner 1997; Schulze *et al.*^2014^). This complex has been implicated in the assembly of IMPs (Botte *et al.*^2016^; Komar *et al.*^2016^). However, mutations affecting each of these components can cause defects in SecA-mediated protein translocation *in vivo* (Gardel *et al.*^1987^, ^1990^; Pogliano and Beckwith 1994; Samuelson *et al.*^2000^), suggesting that the holotranslocon or its individual constituents assist in SecA-mediated translocation.

#### SecDF

In *E. coli*, mutations in the *secD* and *secF* genes cause a strong defect in the translocation of many SecA substrate proteins and a severe cold sensitive growth defect (Gardel *et al.*^1987^, ^1990^). The products of these genes are two IMPs, which form a complex in the cytoplasmic membrane (Gardel *et al.*^1990^; Duong and Wickner 1997). SecD and SecF are encoded in the same operon in *E. coli* and are produced as a single polypeptide chain in many bacteria (Bolhuis *et al.*^1998^). Each protein contains six transmembrane helices, and the pair of proteins resembles complementary halves of a proton-driven pump (Gardel *et al.*^1990^; Tseng *et al.*^1999^). Genetic and biochemical studies suggest that SecDF assists in the later steps of SecA-mediated translocation (Gardel *et al.*^1990^; Nouwen *et al.*^2005^; Tsukazaki *et al.*^2011^). Recent high-resolution structures of SecD and SecF suggest that the large periplasmic domains of these proteins bind to translocating substrate proteins and ratchet them into the periplasm by a mechanism dependent on the PMF (Tsukazaki *et al.*^2011^; Ficici, Jeong and Andricioaei 2017; Furukawa *et al.*^2017^).

#### YajC

In *E. coli*, YajC is encoded in the same polycistronic message as SecD and –F and appears to interact with the SecDF complex (Pogliano and Beckwith 1994; Duong and Wickner 1997). However, the role of YajC in SecA-mediated protein translocation is unknown. However, mutations in the *yajC* gene do not cause a detectable translocation defect on their own (Pogliano and Beckwith 1994).

#### YidC

YidC is a homologue of the Oxa1p in mitochondria and Alb3 in chloroplasts proteins, which are required for protein translocation in these organelles, and depletion of YidC causes a pleiotropic translocation defect in *E. coli* (Samuelson *et al.*^2000^). It has been suggested that YidC promotes the translocation (or insertion) of a distinct subset of IMPs, which do not require SecA or SecYEG for insertion (Froderberg *et al.*^2003^; Celebi *et al.*^2006^; du Plessis, Nouwen and Driessen 2006). It is therefore possible that the defective translocation of SecA substrate proteins in YidC-depletion strains is a secondary consequence of the defective insertion of some other essential protein (Samuelson *et al.*^2000^). However, an uncharacterised mutation (*ssaF*), which is tightly linked to the *yidC* locus, can suppress the temperature sensitivity of a *secA51* mutant (Oliver 1985).

## MATERIALS AND METHODS

The figures given in the subsection ‘**Why do bacteria have a SecA-mediated protein translocation pathway?**’, the graphical abstract and Fig. 1 were derived from the relative protein abundances estimated by Li et al. (2014) from ribosome profiling. Ribosome profiling measures the rate of protein synthesis rather than steady-state abundance and theoretically provides a better basis for estimating the rate of transport of newly synthesised proteins. It is possible that ribosome profiling overestimates the relative abundance of proteins produced at low levels (which could explain the high rate of synthesis estimated for IMPs). However, examination of several protein complexes containing both IMPs and soluble proteins (e.g. NADH reductase, F-ATPase, cytochrome bo oxidase, etc.) suggests that there is not a systematic bias in IMPs produced at similar levels. We estimated the absolute abundance of each protein by multiplying the abundance calculated by Li et al. (2014) by the fraction of the number of ribosomes in an exponentially growing *E. coli* cell (50 000) (Bremer and Dennis 2008) over the abundance of ribosomal protein uL23 estimated by Li et al. (2014) (103 687). OMPs and PPs were identified by searching the UniProtKB database (The UniProt 2017) for *E. coli* K-12 proteins containing the keyword ‘signal peptide’ in the PTM/Processing category, and IMPs were identified by searching UniProtKB for *E. coli* K-12 proteins with the keyword ‘transmembrane’. The length of each protein was determined from its entry in the UniProtKB database (The UniProt 2017).

## CONCLUDING REMARKS

We propose a working model for targeting and translocation by the SecA-mediated translocation pathway based on the literature reviewed here: PPs and OMPs are recognised by the presence of an N-terminal signal sequence and the absence of significant tertiary structure. Substrate proteins are recognised cotranslationally by SecA, but translocation itself is uncoupled from translation and is largely post-translational, possibly resulting from the activity of TF. Alternatively, molecular chaperones can recognise substrate proteins and target them for translocation by interacting with SecA. After delivery to SecYEG, SecA promotes translocation of the proteins across the membrane. Although the mechanism of translocation is not known, it seems clear that translocation must be at least partially probabilistic in order to account for both the large step size and the speed of translocation.

While this model is tidy, it is also incomplete. For example, it does not provide an explanation for the requirement of SecA for SRP-mediated translocation (Schierle *et al.*^2003^) or the binding preference of SecA for nascent IMPs *in vivo* (Huber *et al.*^2017^). In neither case does SecA appear to be responsible for driving translocation since binding of SecA to SecYEG and binding of ribosomes to SecYEG are mutually exclusive (Wu *et al.*^2012^). However, SecA is not thought to be involved in the recognition IMPs since the SRP carries out this step (Saraogi and Shan 2014).

Another question is: How many targeting pathways are there? Recent research suggests SecA can recognise substrate proteins by multiple different mechanisms (Chatzi *et al.*^2017^; Huber *et al.*^2017^; Wang, Yang and Shan 2017). In addition, molecular chaperones could expand the repertoire of the SecA-mediated pathway by specifically recognising a subset of proteins and delivering them to SecA. For example, SecB targets unfolded substrate proteins to SecA under certain conditions (Derman *et al.*^1993^; Fekkes *et al.*^1998^), and overexpression of Hsp70 and GroEL can compensate for translocation defects in some mutant strains of *E. coli* (Phillips and Silhavy 1990; Wild *et al.*^1996^).

Finally, a number of critical questions about the mechanism of translocation still need to be answered: Is SecA-driven translocation processive or probabilistic or a mixture thereof (Bauer *et al.*^2014^; Allen *et al.*^2016^)? If probabilistic, does translocation require mechanical pushing force from SecA (Bauer *et al.*^2014^) or not (Allen *et al.*^2016^)? Is SecA monomeric during translocation *in vivo* (Or, Navon and Rapoport 2002; Or *et al.*^2005^), or does translocation require oligomerisation of SecA (Gouridis *et al.*^2013^)? Similarly, what is the oligomeric state of SecYEG during translocation? Finally, what is the role of the PMF in translocation (Enequist *et al.*^1981^; Schiebel *et al.*^1991^)? Despite over 40 years of research, the Sec machinery continues to provide a rich source of inquiry.

## References

[bib1] Akita M, Shinkai A, Matsuyama S SecA, an essential component of the secretory machinery of *E. coli*, exists as homodimer. Biochem Bioph Res Co 1991;174:211–6. 10.1016/0006-291x(91)90507-4 1824919

[bib2] Allen WJ, Corey RA, Oatley P Two-way communication between SecY and SecA suggests a Brownian ratchet mechanism for protein translocation. eLife 2016;5:pii:e15598. 10.7554/eLife.15598 PMC4907695 27183269

[bib3] Andersson H, Bakker E, von Heijne G Different positively charged amino acids have similar effects on the topology of a polytopic transmembrane protein in *E*. *coli*. J Biol Chem 1992;267:1491–5. 1346135

[bib4] Antonoaea R, Furst M, Nishiyama K The periplasmic chaperone PpiD interacts with secretory proteins exiting from the SecYEG translocon. Biochemistry 2008;47:5649–56. 18439025 10.1021/bi800233w

[bib5] Ariosa A, Lee JH, Wang S Regulation by a chaperone improves substrate selectivity during cotranslational protein targeting. Proc Natl Acad Sci USA 2015;112:E3169–78. 26056263 10.1073/pnas.1422594112 PMC4485088

[bib6] Auclair SM, Moses JP, Musial-Siwek M Mapping of the signal peptide-binding domain of *E. coli* SecA using Forster resonance energy transfer. Biochemistry 2010;49:782–92. 20025247 10.1021/bi901446r PMC2850574

[bib7] Auclair SM, Oliver DB, Mukerji I Defining the solution state dimer structure of *E. coli* SecA using förster resonance energy transfer. Biochemistry 2013;52:2388–401. 23484952 10.1021/bi301217t PMC3717379

[bib8] Baars L, Ytterberg AJ, Drew D Defining the role of the *E. coli* chaperone SecB using comparative proteomics. J Biol Chem 2006;281:10024–34. 16352602 10.1074/jbc.M509929200

[bib9] Banerjee T, Lindenthal C, Oliver D SecA functions *in vivo* as a discrete anti-parallel dimer to promote protein transport. Mol Microbiol 2017;103:439–51. 27802584 10.1111/mmi.13567 PMC5263173

[bib10] Bassford P, Beckwith J *E. coli* mutants accumulating the precursor of a secreted protein in the cytoplasm. Nature 1979;277:538–41. 368649 10.1038/277538a0

[bib11] Bauer BW, Rapoport TA Mapping polypeptide interactions of the SecA ATPase during translocation. Proc Natl Acad Sci USA 2009;106:20800–5. 19933328 10.1073/pnas.0910550106 PMC2780316

[bib12] Bauer BW, Shemesh T, Chen Y A “Push and Slide” mechanism allows sequence-Insensitive translocation of secretory proteins by the SecA ATPase. Cell 2014;157:1416–29. 24906156 10.1016/j.cell.2014.03.063 PMC4104599

[bib13] Beena K, Udgaonkar JB, Varadarajan R Effect of signal peptide on the stability and folding kinetics of maltose binding protein. Biochemistry 2004;43:3608–19. 15035631 10.1021/bi0360509

[bib14] Belin D, Plaia G, Boulfekhar Y *E. coli* SecG is required for residual export mediated by mutant signal sequences and for SecY-SecE complex stability. J Bacteriol 2015;197:542–52. 25404704 10.1128/JB.02136-14 PMC4285974

[bib15] Bieker KL, Phillips GJ, Silhavy TJ Thesec andprl genes of *E. coli*. J Bioenerg Biomembr 1990;22:291–310. 2202721 10.1007/BF00763169

[bib16] Blobel G, Dobberstein B Transfer of proteins across membranes. I. Presence of proteolytically processed and unprocessed nascent immunoglobulin light chains on membrane-bound ribosomes of murine myeloma. J Cell Biol1975a;67:835–51. 811671 10.1083/jcb.67.3.835 PMC2111658

[bib17] Blobel G, Dobberstein B Transfer to proteins across membranes. II. Reconstitution of functional rough microsomes from heterologous components. J Cell Biol1975b;67:852–62. 811672 10.1083/jcb.67.3.852 PMC2111655

[bib18] Bolhuis A, Broekhuizen CP, Sorokin A SecDF of *B**acillus subtilis*, a molecular siamese twin required for the efficient secretion of proteins. J Biol Chem 1998;273:21217–24. 9694879 10.1074/jbc.273.33.21217

[bib19] Bonardi F, Halza E, Walko M Probing the SecYEG translocation pore size with preproteins conjugated with sizable rigid spherical molecules. Proc Natl Acad Sci USA 2011;108:7775–80. 21518907 10.1073/pnas.1101705108 PMC3093497

[bib20] Bornemann T, Holtkamp W, Wintermeyer W Interplay between trigger factor and other protein biogenesis factors on the ribosome. Nat Commun 2014;5:4180. 24939037 10.1038/ncomms5180

[bib21] Botte M, Zaccai NR, Nijeholt JL A central cavity within the holo-translocon suggests a mechanism for membrane protein insertion. Sci Rep 2016;6:38399. 27924919 10.1038/srep38399 PMC5141469

[bib22] Bowers CW, Lau F, Silhavy TJ Secretion of LamB-LacZ by the signal recognition particle pathway of *E. coli*. J Bacteriol 2003;185:5697–705. 13129940 10.1128/JB.185.19.5697-5705.2003 PMC193965

[bib23] Bremer H, Dennis PP Modulation of chemical composition and other parameters of the cell at different exponential growth rates. EcoSal Plus 2008;3, DOI: 10.1128/ecosal.5.2.3. 10.1128/ecosal.5.2.3 26443740

[bib24] Brundage L, Hendrick JP, Schiebel E The purified *E. coli* integral membrane protein SecYE is sufficient for reconstitution of SecA-dependent precursor protein translocation. Cell 1990;62:649–57. 2167176 10.1016/0092-8674(90)90111-q

[bib25] Cannon KS, Or E, Clemons WM Jr Disulfide bridge formation between SecY and a translocating polypeptide localizes the translocation pore to the center of SecY. J Cell Biol 2005;169:219–25. 15851514 10.1083/jcb.200412019 PMC2171872

[bib26] Celebi N, Yi L, Facey SJ Membrane biogenesis of subunit II of cytochrome bo oxidase: contrasting requirements for insertion of N-terminal and C-terminal domains. J Mol Biol 2006;357:1428–36. 16488430 10.1016/j.jmb.2006.01.030

[bib27] Chatzi KE, Sardis MF, Karamanou S Breaking on through to the other side: protein export through the bacterial Sec system. Biochem J 2013;449:25–37. 23216251 10.1042/BJ20121227

[bib28] Chatzi KE, Sardis MF, Tsirigotaki A Preprotein mature domains contain translocase targeting signals that are essential for secretion. J Cell Biol 2017;216:1357–69. 28404644 10.1083/jcb.201609022 PMC5412566

[bib29] Chen Y, Bauer BW, Rapoport TA Conformational changes of the clamp of the protein translocation ATPase SecA. J Mol Biol 2015;427:2348–59. 25982945 10.1016/j.jmb.2015.05.003 PMC4472001

[bib30] Chun SY, Randall LL In vivo studies of the role of SecA during protein export in *E. coli*. J Bacteriol 1994;176:4197–203. 8021205 10.1128/jb.176.14.4197-4203.1994 PMC205629

[bib31] Collier DN, Bankaitis VA, Weiss JB The antifolding activity of SecB promotes the export of the *E. coli* maltose-binding protein. Cell 1988;53:273–83. 2834066 10.1016/0092-8674(88)90389-3

[bib32] Collinson I, Corey RA, Allen WJ Channel crossing: how are proteins shipped across the bacterial plasma membrane? Philos T Roy Soc B 2015;370. 10.1098/rstb.2015.0025 PMC4632601 26370937

[bib33] Das S, Oliver DB Mapping of the SecA·SecY and SecA·SecG interfaces by site-directed *in vivo* photocross-linking. J Biol Chem 2011;286:12371–80. 21317284 10.1074/jbc.M110.182931 PMC3069440

[bib34] den Blaauwen T, Terpetschnig E, Lakowicz JR Interaction of SecB with soluble SecA. FEBS Lett 1997;416:35–38. 9369228 10.1016/s0014-5793(97)01142-3 PMC6939678

[bib35] Derman AI, Puziss JW, Bassford PJ Jr A signal sequence is not required for protein export in prlA mutants of *E. coli*. EMBO J 1993;12:879–88. 8458344 10.1002/j.1460-2075.1993.tb05728.x PMC413286

[bib36] Deville K, Gold VA, Robson A The oligomeric state and arrangement of the active bacterial translocon. J Biol Chem 2011;286:4659–69. 21056980 10.1074/jbc.M110.175638 PMC3039378

[bib37] Doyle SM, Braswell EH, Teschke CM SecA folds via a dimeric intermediate. Biochemistry 2000;39:11667–76. 10995234 10.1021/bi000299y

[bib38] Driessen AJ SecA, the peripheral subunit of the *E. coli* precursor protein translocase, is functional as a dimer. Biochemistry 1993;32:13190–7. 8241173 10.1021/bi00211a030

[bib39] Driessen AJ, Nouwen N Protein translocation across the bacterial cytoplasmic membrane. Annu Rev Biochem 2008;77:643–67. 18078384 10.1146/annurev.biochem.77.061606.160747

[bib40] du Plessis DJ, Nouwen N, Driessen AJ Subunit a of cytochrome o oxidase requires both YidC and SecYEG for membrane insertion. J Biol Chem 2006;281:12248–52. 16513637 10.1074/jbc.M600048200

[bib41] Duong F, Wickner W Distinct catalytic roles of the SecYE, SecG and SecDFyajC subunits of preprotein translocase holoenzyme. EMBO J 1997;16:2756–68. 9184221 10.1093/emboj/16.10.2756 PMC1169885

[bib42] Economou A, Wickner W SecA promotes preprotein translocation by undergoing ATP-driven cycles of membrane insertion and deinsertion. Cell 1994;78:835–43. 8087850 10.1016/s0092-8674(94)90582-7

[bib43] Eisner G, Koch HG, Beck K Ligand crowding at a nascent signal sequence. J Cell Biol 2003;163:35–44. 14530384 10.1083/jcb.200306069 PMC2173441

[bib44] Eisner G, Moser M, Schafer U Alternate recruitment of signal recognition particle and trigger factor to the signal sequence of a growing nascent polypeptide. J Biol Chem 2006;281:7172–9. 16421097 10.1074/jbc.M511388200

[bib45] Emr SD, Schwartz M, Silhavy TJ Mutations altering the cellular localization of the phage lambda receptor, an *E. coli* outer membrane protein. Proc Natl Acad Sci USA 1978;75:5802–6. 104291 10.1073/pnas.75.12.5802 PMC393063

[bib46] Enequist HG, Hirst TR, Harayama S Energy is required for maturation of exported proteins in *E. coli*. Eur J Biochem 1981;116:227–33. 7018904 10.1111/j.1432-1033.1981.tb05323.x

[bib47] Erlandson KJ, Miller SB, Nam Y A role for the two-helix finger of the SecA ATPase in protein translocation. Nature 2008;455:984–7. 18923526 10.1038/nature07439 PMC4354775

[bib48] Eser M, Ehrmann M SecA-dependent quality control of intracellular protein localization. Proc Natl Acad Sci USA 2003;100:13231–4. 14597695 10.1073/pnas.2234410100 PMC263763

[bib49] Fak JJ, Itkin A, Ciobanu DD Nucleotide exchange from the high-affinity ATP-binding site in SecA is the rate-limiting step in the ATPase cycle of the soluble enzyme and occurs through a specialized conformational state. Biochemistry 2004;43:7307–27. 15182175 10.1021/bi0357208

[bib50] Fekkes P, de Wit JG, Boorsma A Zinc stabilizes the SecB binding site of SecA. Biochemistry 1999;38:5111–6. 10213615 10.1021/bi982818r

[bib51] Fekkes P, de Wit JG, van der Wolk JP Preprotein transfer to the *E. coli* translocase requires the co-operative binding of SecB and the signal sequence to SecA. Mol Microbiol 1998;29:1179–90. 9767586 10.1046/j.1365-2958.1998.00997.x

[bib52] Fekkes P, van der Does C, Driessen AJ The molecular chaperone SecB is released from the carboxy-terminus of SecA during initiation of precursor protein translocation. EMBO J 1997;16:6105–13. 9321390 10.1093/emboj/16.20.6105 PMC1326294

[bib53] Ficici E, Jeong D, Andricioaei I Electric-Field-Induced protein translocation via a conformational transition in SecDF: An MD study. Biophys J 2017;112:2520–8. 28636909 10.1016/j.bpj.2017.04.034 PMC5479055

[bib54] Froderberg L, Houben E, Samuelson JC Versatility of inner membrane protein biogenesis in *E. coli*. Mol Microbiol 2003;47:1015–27. 12581356 10.1046/j.1365-2958.2003.03346.x

[bib55] Furukawa A, Yoshikaie K, Mori T Tunnel formation inferred from the I -form structures of the proton-driven protein secretion motor SecDF. Cell Rep 2017;19:895–901. 28467902 10.1016/j.celrep.2017.04.030

[bib56] Gannon PM, Kumamoto CA Mutations of the molecular chaperone protein SecB which alter the interaction between SecB and maltose-binding protein. J Biol Chem 1993;268:1590–5. 8420934

[bib57] Gardel C, Benson S, Hunt J secD, a new gene involved in protein export in *E. coli*. J Bacteriol 1987;169:1286–90. 3029032 10.1128/jb.169.3.1286-1290.1987 PMC211932

[bib58] Gardel C, Johnson K, Jacq A The secD locus of *E. coli* codes for two membrane proteins required for protein export. EMBO J 1990;9:3209–16. 2170107 10.1002/j.1460-2075.1990.tb07519.x PMC552051

[bib59] Gelis I, Bonvin AM, Keramisanou D Structural basis for signal-sequence recognition by the translocase motor SecA as determined by NMR. Cell 2007;131:756–69. 18022369 10.1016/j.cell.2007.09.039 PMC2170882

[bib60] Gold VA, Whitehouse S, Robson A The dynamic action of SecA during the initiation of protein translocation. Biochem J 2013;449:695–705. 23126322 10.1042/BJ20121314 PMC3685266

[bib61] Gouridis G, Karamanou S, Gelis I Signal peptides are allosteric activators of the protein translocase. Nature 2009;462:363–7. 19924216 10.1038/nature08559 PMC2823582

[bib62] Gouridis G, Karamanou S, Sardis MF Quaternary dynamics of the SecA motor drive translocase catalysis. Mol Cell 2013;52:655–66. 24332176 10.1016/j.molcel.2013.10.036

[bib63] Govindarajan S, Amster-Choder O The bacterial Sec system is required for the organization and function of the MreB cytoskeleton. PLoS Genet 2017;13:e1007017. 28945742 10.1371/journal.pgen.1007017 PMC5629013

[bib64] Gumbart J, Schulten K Molecular dynamics studies of the archaeal translocon. Biophys J 2006;90:2356–67. 16415058 10.1529/biophysj.105.075291 PMC1403164

[bib65] Gumbart J, Schulten K Structural determinants of lateral gate opening in the protein translocon. Biochemistry 2007;46:11147–57. 17760424 10.1021/bi700835d

[bib66] Hartl FU, Lecker S, Schiebel E The binding cascade of SecB to SecA to SecYE mediates preprotein targeting to the *E. coli* plasma membrane. Cell 1990;63:269–79. 2170023 10.1016/0092-8674(90)90160-g

[bib67] Hatahet F, Boyd D, Beckwith J Disulfide bond formation in prokaryotes: history, diversity and design. BBA- Proteins Proteom 2014;1844:1402–14. 10.1016/j.bbapap.2014.02.014 PMC4048783 24576574

[bib68] Hegde RS, Bernstein HD The surprising complexity of signal sequences. Trends Biochem Sci 2006;31:563–71. 16919958 10.1016/j.tibs.2006.08.004

[bib69] Hirano M, Matsuyama S, Tokuda H The carboxyl-terminal region is essential for Sec-A dimerization. Biochem Bioph Res Co 1996;229:90–5. 10.1006/bbrc.1996.1762 8954088

[bib70] Hizlan D, Robson A, Whitehouse S Structure of the SecY complex unlocked by a preprotein mimic. Cell Rep 2012;1:21–28. 22576621 10.1016/j.celrep.2011.11.003 PMC3333808

[bib71] Huber D, Boyd D, Xia Y Use of thioredoxin as a reporter to identify a subset of *E. coli* signal sequences that promote signal recognition particle-dependent translocation. J Bacteriol2005a;187:2983–91. 15838024 10.1128/JB.187.9.2983-2991.2005 PMC1082830

[bib72] Huber D, Cha MI, Debarbieux L A selection for mutants that interfere with folding of *E. coli* thioredoxin-1 in vivo. Proc Natl Acad Sci USA2005b;102:18872–7. 16357193 10.1073/pnas.0509583102 PMC1323206

[bib73] Huber D, Chaffotte A, Eser M Amino acid residues important for folding of thioredoxin are revealed only by study of the physiologically relevant reduced form of the protein. Biochemistry 2010;49:8922–8. 20873718 10.1021/bi100784h PMC2953564

[bib74] Huber D, Jamshad M, Hanmer R SecA cotranslationally interacts with nascent substrate proteins *In Vivo*. J Bacteriol 2017;199:pii:e00622–16. 27795329 10.1128/JB.00622-16 PMC5198489

[bib75] Huber D, Rajagopalan N, Preissler S SecA interacts with ribosomes in order to facilitate posttranslational translocation in bacteria. Mol Cell 2011;41:343–53. 21292166 10.1016/j.molcel.2010.12.028

[bib76] Hunt JF, Weinkauf S, Henry L Nucleotide control of interdomain interactions in the conformational reaction cycle of SecA. Science 2002;297:2018–26. 12242434 10.1126/science.1074424

[bib77] Jilaveanu LB, Oliver D SecA dimer cross-linked at its subunit interface is functional for protein translocation. J Bacteriol 2006;188:335–8. 16352850 10.1128/JB.188.1.335-338.2006 PMC1317605

[bib78] Jilaveanu LB, Zito CR, Oliver D Dimeric SecA is essential for protein translocation. Proc Natl Acad Sci USA 2005;102:7511–6. 15897468 10.1073/pnas.0502774102 PMC1140455

[bib79] Josefsson LG, Randall LL Different exported proteins in E. coli show differences in the temporal mode of processing in vivo. Cell1981a;25:151–7. 7023693 10.1016/0092-8674(81)90239-7

[bib80] Josefsson LG, Randall LL Processing in vivo of precursor maltose-binding protein in *E. coli* occurs post-translationally as well as co-translationally. J Biol Chem1981b;256:2504–7. 7007385

[bib81] Kadokura H, Beckwith J Detecting folding intermediates of a protein as it passes through the bacterial translocation channel. Cell 2009;138:1164–73. 19766568 10.1016/j.cell.2009.07.030 PMC2750780

[bib82] Kebir MO, Kendall DA SecA specificity for different signal peptides. Biochemistry 2002;41:5573–80. 11969418 10.1021/bi015798t

[bib83] Knoblauch NT, Rudiger S, Schonfeld HJ Substrate specificity of the SecB chaperone. J Biol Chem 1999;274:34219–25. 10567394 10.1074/jbc.274.48.34219

[bib84] Komar J, Alvira S, Schulze RJ Membrane protein insertion and assembly by the bacterial holo-translocon SecYEG-SecDF-YajC-YidC. Biochem J 2016;473:3341–54. 27435098 10.1042/BCJ20160545 PMC5095914

[bib85] Krehenbrink M, Edwards A, Downie JA The superoxide dismutase SodA is targeted to the periplasm in a SecA-dependent manner by a novel mechanism. Mol Microbiol 2011;82:164–79. 21854464 10.1111/j.1365-2958.2011.07803.x

[bib86] Kumamoto CA, Beckwith J Mutations in a new gene, secB, cause defective protein localization in *E. coli*. J Bacteriol 1983;154:253–60. 6403503 10.1128/jb.154.1.253-260.1983 PMC217454

[bib87] Kumamoto CA, Beckwith J Evidence for specificity at an early step in protein export in *E. coli*. J Bacteriol 1985;163:267–74. 3891730 10.1128/jb.163.1.267-274.1985 PMC219108

[bib88] Kumamoto CA, Francetic O Highly selective binding of nascent polypeptides by an *E. coli* chaperone protein in vivo. J Bacteriol 1993;175:2184–8. 8468278 10.1128/jb.175.8.2184-2188.1993 PMC204502

[bib89] Kumamoto CA, Gannon PM Effects of *E. coli* secB mutations on pre-maltose binding protein conformation and export kinetics. J Biol Chem 1988;263:11554–8. 3042772

[bib90] Kusters I, van den Bogaart G, Kedrov A Quaternary structure of SecA in solution and bound to SecYEG probed at the single molecule level. Structure 2011;19:430–9. 21397193 10.1016/j.str.2010.12.016

[bib91] Lee CA, Beckwith J Suppression of growth and protein secretion defects in *E. coli* secA mutants by decreasing protein synthesis. J Bacteriol 1986;166:878–83. 3519584 10.1128/jb.166.3.878-883.1986 PMC215208

[bib92] Lee HC, Bernstein HD The targeting pathway of *E. coli* presecretory and integral membrane proteins is specified by the hydrophobicity of the targeting signal. Proc Natl Acad Sci USA 2001;98:3471–6. 11248102 10.1073/pnas.051484198 PMC30677

[bib93] Lee HC, Bernstein HD Trigger factor retards protein export in *E. coli*. J Biol Chem 2002;277:43527–35. 12205085 10.1074/jbc.M205950200

[bib94] Li GW, Burkhardt D, Gross C Quantifying absolute protein synthesis rates reveals principles underlying allocation of cellular resources. Cell 2014;157:624–35. 24766808 10.1016/j.cell.2014.02.033 PMC4006352

[bib95] Li L, Park E, Ling J Crystal structure of a substrate-engaged SecY protein-translocation channel. Nature 2016;531:395–9. 26950603 10.1038/nature17163 PMC4855518

[bib96] Li W, Schulman S, Boyd D The plug domain of the SecY protein stabilizes the closed state of the translocation channel and maintains a membrane seal. Mol Cell 2007;26:511–21. 17531810 10.1016/j.molcel.2007.05.002

[bib97] Lill R, Cunningham K, Brundage LA SecA protein hydrolyzes ATP and is an essential component of the protein translocation ATPase of *E. coli*. Embo J 1989;8:961–6. 2542029 10.1002/j.1460-2075.1989.tb03458.x PMC400897

[bib98] Liu GP, Topping TB, Cover WH Retardation of folding as a possible means of suppression of a mutation in the leader sequence of an exported protein. J Biol Chem 1988;263:14790–3. 3049590

[bib99] Matsuyama S, Akimaru J, Mizushima S SecE-dependent overproduction of SecY in *E. coli*. FEBS Lett 1990;269:96–100. 2201574 10.1016/0014-5793(90)81128-b

[bib100] Mori H, Ito K Different modes of SecY-SecA interactions revealed by site-directed in vivo photo-cross-linking. Proc Natl Acad Sci USA 2006;103:16159–64. 17060619 10.1073/pnas.0606390103 PMC1621050

[bib101] Mori H, Tsukazaki T, Masui R Fluorescence resonance energy transfer analysis of protein translocase. J Biol Chem 2003;278:14257–64. 12533543 10.1074/jbc.M300230200

[bib102] Nishiyama K, Hanada M, Tokuda H Disruption of the gene encoding p12 (SecG) reveals the direct involvement and important function of SecG in the protein translocation of *E. coli* at low temperature. EMBO J 1994;13:3272–7. 8045257 10.1002/j.1460-2075.1994.tb06628.x PMC395223

[bib103] Nouwen N, Piwowarek M, Berrelkamp G The large first periplasmic loop of SecD and SecF plays an important role in SecDF functioning. J Bacteriol 2005;187:5857–60. 16077136 10.1128/JB.187.16.5857-5860.2005 PMC1196081

[bib104] Oh E, Becker AH, Sandikci A Selective ribosome profiling reveals the cotranslational chaperone action of trigger factor *in vivo*. Cell 2011;147:1295–308. 22153074 10.1016/j.cell.2011.10.044 PMC3277850

[bib105] Oliver DB Identification of five new essential genes involved in the synthesis of a secreted protein in *E. coli*. J Bacteriol 1985;161:285–91. 3881390 10.1128/jb.161.1.285-291.1985 PMC214869

[bib106] Oliver DB, Beckwith J *E. coli* mutant pleiotropically defective in the export of secreted proteins. Cell 1981;25:765–72. 7026050 10.1016/0092-8674(81)90184-7

[bib107] Oliver DB, Beckwith J Identification of a new gene (secA) and gene product involved in the secretion of envelope proteins in *E. coli*. J Bacteriol1982a;150:686–91. 6279567 10.1128/jb.150.2.686-691.1982 PMC216417

[bib108] Oliver DB, Beckwith J Regulation of a membrane component required for protein secretion in *E. coli*. Cell1982b;30:311–9. 6751561 10.1016/0092-8674(82)90037-x

[bib109] Or E, Boyd D, Gon S The bacterial ATPase SecA functions as a monomer in protein translocation. J Biol Chem 2005;280:9097–105. 15618215 10.1074/jbc.M413947200

[bib110] Or E, Navon A, Rapoport T Dissociation of the dimeric SecA ATPase during protein translocation across the bacterial membrane. EMBO J 2002;21:4470–9. 12198149 10.1093/emboj/cdf471 PMC126201

[bib111] Osborne AR, Rapoport TA Protein translocation is mediated by oligomers of the SecY complex with one SecY copy forming the channel. Cell 2007;129:97–110. 17418789 10.1016/j.cell.2007.02.036

[bib112] Papanikolau Y, Papadovasilaki M, Ravelli RB Structure of dimeric SecA, the *E. coli* preprotein translocase motor. J Mol Biol 2007;366:1545–57. 17229438 10.1016/j.jmb.2006.12.049

[bib113] Park E, Rapoport TA Mechanisms of Sec61/SecY-mediated protein translocation across membranes. Annu Rev Biophys 2012;41:21–40. 22224601 10.1146/annurev-biophys-050511-102312

[bib114] Park S, Liu G, Topping TB Modulation of folding pathways of exported proteins by the leader sequence. Science 1988;239:1033–5. 3278378 10.1126/science.3278378

[bib115] Perlman D, Halvorson HO A putative signal peptidase recognition site and sequence in eukaryotic and prokaryotic signal peptides. J Mol Biol 1983;167:391–409. 6345794 10.1016/s0022-2836(83)80341-6

[bib116] Pettersen EF, Goddard TD, Huang CC UCSF Chimera–a visualization system for exploratory research and analysis. J Comput Chem 2004;25:1605–12. 15264254 10.1002/jcc.20084

[bib117] Phillips GJ, Silhavy TJ Heat-shock proteins DnaK and GroEL facilitate export of LacZ hybrid proteins in E. coli. Nature 1990;344:882–4. 2109835 10.1038/344882a0

[bib118] Pogliano KJ, Beckwith J Genetic and molecular characterization of the *E. coli* secD operon and its products. J Bacteriol 1994;176:804–14. 7507921 10.1128/jb.176.3.804-814.1994 PMC205118

[bib119] Prinz WA, Spiess C, Ehrmann M Targeting of signal sequenceless proteins for export in *E. coli* with altered protein translocase. EMBO J 1996;15:5209–17. 8895566 PMC452265

[bib120] Pugsley AP The complete general secretory pathway in gram-negative bacteria. Microbiol Rev 1993;57:50–108. 8096622 10.1128/mr.57.1.50-108.1993 PMC372901

[bib121] Randall LL Translocation of domains of nascent periplasmic proteins across the cytoplasmic membrane is independent of elongation. Cell 1983;33:231–40. 6380753 10.1016/0092-8674(83)90352-5

[bib122] Randall LL, Hardy SJ Correlation of competence for export with lack of tertiary structure of the mature species: a study in vivo of maltose-binding protein in *E. coli*. Cell 1986;46:921–8. 3530497 10.1016/0092-8674(86)90074-7

[bib123] Randall LL, Hardy SJ SecB, one small chaperone in the complex milieu of the cell. Cell Mol Life Sci 2002;59:1617–23. 12475171 10.1007/PL00012488 PMC11337512

[bib124] Randall LL, Henzl MT Direct identification of the site of binding on the chaperone SecB for the amino terminus of the translocon motor SecA. Protein Sci 2010;19:1173–9. 20512970 10.1002/pro.392 PMC2895241

[bib125] Randall LL, Topping TB, Hardy SJ Binding of SecB to ribosome-bound polypeptides has the same characteristics as binding to full-length, denatured proteins. Proc Natl Acad Sci USA 1997;94:802–7. 9023337 10.1073/pnas.94.3.802 PMC19594

[bib126] Randall LL, Topping TB, Suciu D Calorimetric analyses of the interaction between SecB and its ligands. Protein Sci 1998;7:1195–200. 9605324 10.1002/pro.5560070514 PMC2144013

[bib127] Sachelaru I, Petriman NA, Kudva R YidC occupies the lateral gate of the SecYEG translocon and is sequentially displaced by a nascent membrane protein. J Biol Chem 2013;288:16295–307. 23609445 10.1074/jbc.M112.446583 PMC3675568

[bib128] Sachelaru I, Petriman NA, Kudva R Dynamic interaction of the sec translocon with the chaperone PpiD. J Biol Chem 2014;289:21706–15. 24951590 10.1074/jbc.M114.577916 PMC4118129

[bib129] Samuelson JC, Chen M, Jiang F YidC mediates membrane protein insertion in bacteria. Nature 2000;406:637–41. 10949305 10.1038/35020586

[bib130] Saraogi I, Shan SO Co-translational protein targeting to the bacterial membrane. BBA-Mol Cell Res 2014;1843:1433–41. 10.1016/j.bbamcr.2013.10.013 PMC3999308 24513458

[bib131] Schatz G, Dobberstein B Common principles of protein translocation across membranes. Science 1996;271:1519–26. 8599107 10.1126/science.271.5255.1519

[bib132] Schibich D, Gloge F, Pohner I Global profiling of SRP interaction with nascent polypeptides. Nature 2016;536:219–23. 27487212 10.1038/nature19070

[bib133] Schiebel E, Driessen AJ, Hartl FU Delta mu H+ and ATP function at different steps of the catalytic cycle of preprotein translocase. Cell 1991;64:927–39. 1825804 10.1016/0092-8674(91)90317-r

[bib134] Schierle CF, Berkmen M, Huber D The DsbA signal sequence directs efficient, cotranslational export of passenger proteins to the *E. coli* Periplasm via the signal recognition particle pathway. J Bacteriol 2003;185:5706–13. 13129941 10.1128/JB.185.19.5706-5713.2003 PMC193964

[bib135] Schmidt MG, Rollo EE, Grodberg J Nucleotide sequence of the secA gene and secA(Ts) mutations preventing protein export in *E. coli*. J Bacteriol 1988;170:3404–14. 2841285 10.1128/jb.170.8.3404-3414.1988 PMC211308

[bib136] Schulze RJ, Komar J, Botte M Membrane protein insertion and proton-motive-force-dependent secretion through the bacterial holo-translocon SecYEG-SecDF-YajC-YidC. Proc Natl Acad Sci USA 2014;111:4844–9. 24550475 10.1073/pnas.1315901111 PMC3977283

[bib137] Sharma V, Arockiasamy A, Ronning DR Crystal structure of *Mycobacterium tuberculosis* SecA, a preprotein translocating ATPase. Proc Natl Acad Sci USA 2003;100:2243–8. 12606717 10.1073/pnas.0538077100 PMC151325

[bib138] Shimizu H, Nishiyama K, Tokuda H Expression of gpsA encoding biosynthetic sn-glycerol 3-phosphate dehydrogenase suppresses both the LB? phenotype of a secB null mutant and the cold-sensitive phenotype of a secG null mutant. Mol Microbiol 1997;26:1013–21. 9426138 10.1046/j.1365-2958.1997.6392003.x

[bib139] Simon SM, Peskin CS, Oster GF What drives the translocation of proteins? Proc Natl Acad Sci USA 1992;89:3770–4. 1349170 10.1073/pnas.89.9.3770 PMC525572

[bib140] Singh R, Kraft C, Jaiswal R Cryo-electron microscopic structure of SecA protein bound to the 70S ribosome. J Biol Chem 2014;289:7190–9. 24443566 10.1074/jbc.M113.506634 PMC3945378

[bib141] Song T, Park C Effect of folding on the export of ribose-binding protein studied with the genetically isolated suppressors for the signal sequence mutation. J Mol Biol 1995;253:304–12. 7563091 10.1006/jmbi.1995.0554

[bib142] Steiner D, Forrer P, Stumpp MT Signal sequences directing cotranslational translocation expand the range of proteins amenable to phage display. Nat Biotechnol 2006;24:823–31. 16823375 10.1038/nbt1218

[bib143] Tani K, Shiozuka K, Tokuda H In vitro analysis of the process of translocation of OmpA across the *E. coli* cytoplasmic membrane. A translocation intermediate accumulates transiently in the absence of the proton motive force. J Biol Chem 1989;264:18582–8. 2553715

[bib144] Taura T, Baba T, Akiyama Y Determinants of the quantity of the stable SecY complex in the *E. coli* cell. J Bacteriol 1993;175:7771–5. 8253665 10.1128/jb.175.24.7771-7775.1993 PMC206951

[bib145] Teschke CM, Kim J, Song T Mutations that affect the folding of ribose-binding protein selected as suppressors of a defect in export in *E. coli*. J Biol Chem 1991;266:11789–96. 1904869

[bib146] The UniProt C UniProt: the universal protein knowledgebase. Nucleic Acids Res 2017;45:D158–69. 27899622 10.1093/nar/gkw1099 PMC5210571

[bib147] Tian P, Andricioaei I Size, motion, and function of the SecY translocon revealed by molecular dynamics simulations with virtual probes. Biophys J 2006;90:2718–30. 16461399 10.1529/biophysj.105.073304 PMC1414555

[bib148] Tomkiewicz D, Nouwen N, van Leeuwen R SecA supports a constant rate of preprotein translocation. J Biol Chem 2006;281:15709–13. 16601117 10.1074/jbc.M600205200

[bib149] Tseng TT, Gratwick KS, Kollman J The RND permease superfamily: an ancient, ubiquitous and diverse family that includes human disease and development proteins. J Mol Microb Biotech 1999;1:107–25. 10941792

[bib150] Tsukazaki T, Mori H, Echizen Y Structure and function of a membrane component SecDF that enhances protein export. Nature 2011;474:235–8. 21562494 10.1038/nature09980 PMC3697915

[bib151] Uchida K, Mori H, Mizushima S Stepwise movement of preproteins in the process of translocation across the cytoplasmic membrane of *E. coli*. J Biol Chem 1995;270:30862–8. 8537339 10.1074/jbc.270.52.30862

[bib152] Ulbrandt ND, Newitt JA, Bernstein HD The *E. coli* signal recognition particle is required for the insertion of a subset of inner membrane proteins. Cell 1997;88:187–96. 9008159 10.1016/s0092-8674(00)81839-5

[bib153] Ullers RS, Ang D, Schwager F Trigger Factor can antagonize both SecB and DnaK/DnaJ chaperone functions in *E. coli*. Proc Natl Acad Sci USA 2007;104:3101–6. 17360615 10.1073/pnas.0608232104 PMC1805596

[bib154] Van den Berg B, Clemons WM Jr, Collinson I X-ray structure of a protein-conducting channel. Nature 2004;427:36–44. 14661030 10.1038/nature02218

[bib155] van der Sluis EO, Driessen AJ Stepwise evolution of the sec machinery in Proteobacteria. Trends Microbiol 2006;14:105–8. 16490356 10.1016/j.tim.2006.01.009

[bib156] van der Wolk JP, de Wit JG, Driessen AJ The catalytic cycle of the *E. coli* SecA ATPase comprises two distinct preprotein translocation events. EMBO J 1997;16:7297–304. 9405359 10.1093/emboj/16.24.7297 PMC1170330

[bib157] van Stelten J, Silva F, Belin D Effects of antibiotics and a proto-oncogene homolog on destruction of protein translocator SecY. Science 2009;325:753–6. 19661432 10.1126/science.1172221 PMC2832214

[bib158] Vassylyev DG, Mori H, Vassylyeva MN Crystal structure of the translocation ATPase SecA from *Thermus thermophilus* reveals a parallel, head-to-head dimer. J Mol Biol 2006;364:248–58. 17059823 10.1016/j.jmb.2006.09.061

[bib159] Veenendaal AK, van der Does C, Driessen AJ Mapping the sites of interaction between SecY and SecE by cysteine scanning mutagenesis. J Biol Chem 2001;276:32559–66. 11445571 10.1074/jbc.M103912200

[bib160] von Heijne G The signal peptide. J Membrain Biol 1990;115:195–201. 10.1007/BF01868635 2197415

[bib161] Wang M, Herrmann CJ, Simonovic M Version 4.0 of PaxDb: protein abundance data, integrated across model organisms, tissues, and cell-lines. Proteomics 2015;15:3163–8. 25656970 10.1002/pmic.201400441 PMC6680238

[bib162] Wang S, Yang CI, Shan SO SecA mediates cotranslational targeting and translocation of an inner membrane protein. J Cell Biol 2017;216:3639–53. 28928132 10.1083/jcb.201704036 PMC5674894

[bib163] Whitehouse S, Gold VA, Robson A Mobility of the SecA 2-helix-finger is not essential for polypeptide translocation via the SecYEG complex. J Cell Biol 2012;199:919–29. 23209305 10.1083/jcb.201205191 PMC3518217

[bib164] Wild J, Rossmeissl P, Walter WA Involvement of the DnaK-DnaJ-GrpE chaperone team in protein secretion in *E. coli*. J Bacteriol 1996;178:3608–13. 8655561 10.1128/jb.178.12.3608-3613.1996 PMC178133

[bib165] Wolfe PB, Wickner W Bacterial leader peptidase, a membrane protein without a leader peptide, uses the same export pathway as pre-secretory proteins. Cell 1984;36:1067–72. 6368003 10.1016/0092-8674(84)90056-4

[bib166] Woodbury RL, Hardy SJ, Randall LL Complex behavior in solution of homodimeric SecA. Protein Sci 2002;11:875–82. 11910030 10.1110/ps.4090102 PMC2373524

[bib167] Wu ZC, de Keyzer J, Kedrov A Competitive binding of the SecA ATPase and ribosomes to the SecYEG translocon. J Biol Chem 2012;287:7885–95. 22267723 10.1074/jbc.M111.297911 PMC3318685

[bib168] Ye J, Osborne AR, Groll M RecA-like motor ATPases–lessons from structures. BBA- Bioenergetics 2004;1659:1–18. 15511523 10.1016/j.bbabio.2004.06.003

[bib169] Zhang Q, Li Y, Olson R Conserved SecA signal peptide-binding site revealed by engineered protein chimeras and Förster resonance energy transfer. Biochemistry 2016;55:1291–300. 26854513 10.1021/acs.biochem.5b01115 PMC4883009

[bib170] Zimmer J, Li W, Rapoport TA A novel dimer interface and conformational changes revealed by an X-ray structure of B. subtilis SecA. J Mol Biol 2006;364:259–65. 16989859 10.1016/j.jmb.2006.08.044

[bib171] Zimmer J, Nam Y, Rapoport TA Structure of a complex of the ATPase SecA and the protein-translocation channel. Nature 2008;455:936–43. 18923516 10.1038/nature07335 PMC7164768

[bib172] Zimmer J, Rapoport TA Conformational flexibility and peptide interaction of the translocation ATPase SecA. J Mol Biol 2009;394:606–12. 19850053 10.1016/j.jmb.2009.10.024 PMC2832196

